# Phase transitions and (*p–T–X*) behaviour of centrosymmetric perovskites: modelling with transformed crystallographic data

**DOI:** 10.1107/S2052520621012713

**Published:** 2022-01-20

**Authors:** Noel W. Thomas

**Affiliations:** a Werkstofftechnik Glas and Keramik, Hochschule Koblenz, Rheinstrasse 56, 56203 Hoehr-Grenzhausen, Germany

**Keywords:** perovskite, centrosymmetric, phase transition, *p–T–X*, octahedral distortion, octahedral tilting, structural modelling

## Abstract

A reversible transformation of the unit-cell parameters and atomic coordinates of centrosymmetric perovskites *ABX*_3_ into a Cartesian space is defined. Analytical expressions for the three vectors for the pseudocubic cell and three vectors for a *BX*_6_ octahedron are derived for space groups *Pbmn*, C*mcm*, *Ibmm*, *P*4/*mbm*, *P*4/*nmc*, *I*4/*mcm* and *R* 3 *c*. The following structural parameters may be derived from these vectors: up to six pseudocubic parameters defining octahedral geometry; length- and angle-based octahedral distortion parameters λ and σ; inclination angles of tilted octahedra, θ_1_, θ_2_ and θ_3_; angles of tilt of octahedra; *AX*_12_:*BX*_6_ polyhedral volume ratio, *V*_*A*_/*V*_*B*_; parameters η_*A*_ and η_*B*_ defining the relative contraction of inner *AX*_8_ polyhedra and expansion of *BX*_6_ octahedra due to octahedral tilting. The application of these parameters is demonstrated by reference to published crystal structures. The variation of η_*A*_ and η_*B*_ with temperature in the compositional series Sr*_x_* Ba_1–*x*_SnO_3_ and Sr*_x_* Ba_1–*x*_HfO_3_, as well as the temperature series of BaPbO_3_ and CaTiO_3_, is related to the sequence of phases *Pbmn* → *Ibmm*→ *Pm* 3 *m*. Stabilization of the C*mcm* phase is likewise interpreted in terms of these two parameters for NaTaO_3_ and NaNbO_3_. The pressure evolution of the structures of MgSiO_3_, YAlO_3_, (La_1–*x*_Nd*_x_*)GaO_3_ (0 ≤ *x* ≤ 1) and YAl_0.25_Cr_0.75_O_3_ is modelled with the appropriate structural parameters, thereby also addressing the characteristics of the *Pbmn* → *R* 3 *c* transition. Simulation of MgSiO_3_ up to 125 GPa and of YAlO_3_ up to 52 GPa in space group *Pbnm* is carried out by using the Birch–Murnaghan equation of state. In both cases, full sets of oxygen coordinates assuming regular octahedra are generated. Octahedral distortion is also modelled in the latter system and predicted to have a key influence on structural evolution and the sequence of phase transitions. The core modelling procedures are made available as a Microsoft *Excel* file.

## Abstract 2

The structures of centrosymmetric perovskites (*ABX*_3_) are modelled by transforming crystallographic data into a Cartesian space defining *BX*_6_ octahedral tilting and distortion. Structural evolution and phase transitions under variable (*p–T–X*)-conditions are analysed and predicted.

## 1. Introduction: the modelling of octahedral tilting in perovskites

Synthetic perovskite-related compounds continue to attract the attention of many scientists and technologists, irrespective of whether they are working, for example, on the development of lead-free piezoelectric ceramics (Shrout & Zhang, 2007 ▸) or on organolead halide *ABX*_3_ nanocrystals for photochemical cells (Jena *et al.*, 2019 ▸). In general, the current availability of high-quality structural data evokes the need for all the nuances of structural change under varying conditions of pressure, temperature and composition] (*p–T–X*) to be precisely modelled. This expectation is particularly poignant in the case of perovskite-related compounds, *ABX*_3_, since, according to Mitchell (2002*a* ▸), all elements, apart from the noble gases, can be found in a variant of these. In the pioneering work of Megaw (1973 ▸), their structures were regarded as comprising three essential features: (*a*) tilting of the anion octahedra; (*b*) displacements of the cations; (*c*) octahedral distortions. Definitive work by Glazer (1972 ▸) led to a system of classification based on the sense of tilt of the three tetrad axes of corner-linked regular octahedra about three perpendicular, or nearly perpendicular crystal axes.

**Figure 1:**
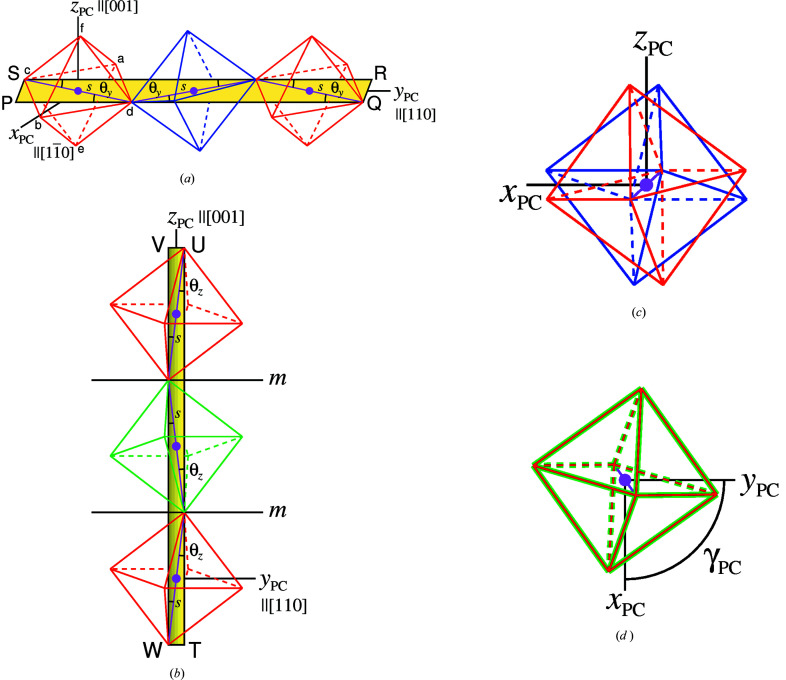
Tilting of regular octahedra about the *x*_PC_ and *y*_PC_ axes, as in space group *Pbnm*. Pseudocubic axes *x*_PC_, *y*_PC_ and *z*_PC_ are directed parallel to orthorhombic vectors 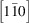 , 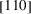 and [001], respectively. (*a*) Tilting around the *y*_PC_ axis (in clinographic projection) with θ*_y_* the angles of inclination of stalks of length *s* to the axis; (*b*) Tilting around the *z*_PC_ axis (head-on view showing mirror planes); (*c*) *y*_PC_-axis tilting [as in (*a*)] viewed along the *y*_PC_ axis towards the origin; (*d*) *z*_PC_-axis tilting [as in (*b*)] viewed along the *z*_PC_ axis towards the origin.

A regular octahedron is characterized by three pairs of opposite vertices that are linked by stalks of equal length passing through its mid-point at right angles to one another. Two lines of regular octahedra are shown in Figs. 1 ▸(*a*) and 1 ▸(*b*), as they arise in space group *Pbnm*.

Zigzag chains of adjacent octahedral stalks of uniform length *s* are formed, these lying in planes PQRS and TUVW. When viewed along the *y*_PC_ axis-of-tilting, the red and blue octahedra are seen to be rotated in opposite senses [Fig. 1 ▸(*c*)], leading to Glazer notation 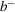 . By comparison, the red and green octahedra are rotated in the same sense [Fig. 1 ▸(*d*)]. This leads to the notation 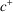 , since the tilt angle is different. As tilting around the *x*_PC_ and *y*_PC_ axes is equivalent, the three-dimensional tilt system in *Pbnm* is denoted by 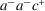 (Glazer, 1972 ▸).

**Figure 2:**
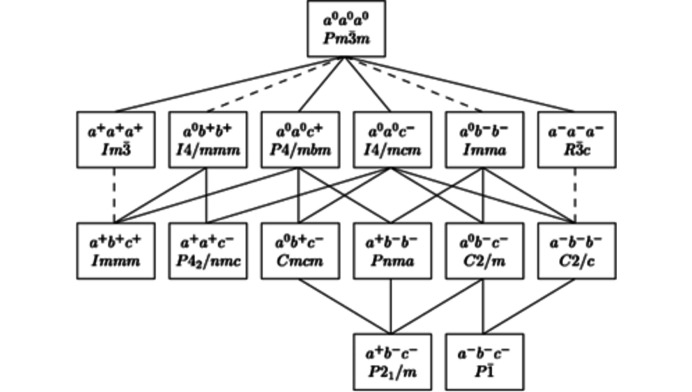
Group–subgroup relationships among the 15 perovskite space groups arising from the tilting of regular octahedra. A dashed line signifies that the corresponding phase transition must be first order (Howard & Stokes, 2002 ▸). Reproduced with permission of the International Union of Crystallography.

In this now well established *a* ^#^ *b* ^#^ *c* ^#^ nomenclature, superscripts # can be +, − or 0, denoting the angles of tilt of neighbouring octahedra along the three pseudocubic axes as in-phase (+), anti-phase (−) or zero. Importantly, Glazer (1972 ▸) also showed that the 23 tilt systems for regular octahedra correlated with 15 alternative space groups. As well as aiding the correct interpretation of perovskite diffraction patterns (Glazer, 1975 ▸), these correlations stimulated group-theoretical analysis of perovskites, their being confirmed significantly later by Howard & Stokes (1998 ▸, 2002 ▸). The latter work led to minor modifications to the space groups originally assigned by Glazer and established a top-down, hierarchical system of group–subgroup relationships starting from the cubic 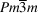 aristotype for perovskite structures with tilted, regular octahedra (Fig. 2 ▸).

Two independent contributions concerning octahedral tilting in perovskites were made by Woodward (1997*a* ▸,*b* ▸) and Thomas (1996 ▸). Woodward (1997*a* ▸) concluded that perfectly regular octahedra could not be linked together in some tilt systems. The contentious space groups were subsequently narrowed down to *Cmcm* (tilt system 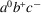 ) and *P*4_2_/*nmc* ( 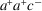 ) by Howard & Stokes (1998 ▸), with only the latter requiring irregular octahedra. The work of Thomas (1996 ▸), by comparison, was primarily concerned with the dependence of *AX*_12_:*BX*_6_ polyhedral volume ratio, *V_A_/V_B_*, on octahedral tilting in orthorhombic and tetragonal perovskites. Inclination angles θ*_y_* and θ*_z_* in Figs. 1 ▸(*a*) and 1 ▸(*b*), and by extension θ*_x_*, were used to relate octahedral stalk lengths *s* and tilting to the lengths of pseudocubic cell axes 1, 2 and 3 and therefore cell volume [equation (1)].

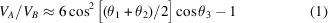

This form results from the coupling of inclination angles θ_1_ and θ_2_ when pseudocubic axes 1 and 2 are oriented at approximately 45° to the crystal axes. In untilted structures (with θ_1_ = θ_2_ = θ_3_ = 0), *V_A_/V_B_* is exactly equal to five (Thomas, 1989 ▸).

Tamazyan & van Smaalen (2007 ▸) subsequently argued that inclination angles θ_1_, θ_2_ and θ_3_ defined by Thomas (1996 ▸) were unnecessarily influenced by octahedral distortion. They therefore proposed an alternative method of calculating tilt angles, although this was at the expense of losing the simple link to polyhedral volume ratio expressed by equation (1). Later work by Wang & Angel (2011 ▸) restored the link between octahedral tilting and ratio *V_A_/V_B_*, employing a group-theoretical, rather than a geometrical approach to separate octahedral tilting and distortion. These authors expressed the ratio *V_A_/V_B_* as a function of the amplitudes of the normal modes in a cubic perovskite, rather than by direct calculation from anion coordinates in experimentally determined crystal structures.

The conflict between geometrical, *i.e.* crystal-chemical and group-theoretical methods in describing perovskite structures is somewhat contrived, since both have legitimate fields of application and ultimately have similar aims. This was made clear in seminal work by Knight (2009 ▸), who utilized group-theoretical methods to develop a general parameterization of centrosymmetric perovskites based on symmetry-adapted basis vectors of the 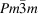 phase. He also pointed to difficulties with a method earlier proposed by this author for a general crystal-chemical parameterization of centrosymmetric perovskites (Thomas, 1998 ▸). Two specific problems were identified, (*a*) that the method was ‘geometrically complex’; and (*b*) that it relied ‘totally on an empirical analysis of known crystal structures’. He also made the important point, (*c*), that the *A* site has a coordination number less than 12 due to a geometrically complex coordination polyhedron (Knight, 2009 ▸). In the current work, point (*a*) is addressed by focusing on the distortion of centrosymmetric octahedra as independent geometrical forms. Point (*b*) is addressed by deriving analytical expressions dependent on space group for the three stalk vectors defining octahedral geometry. Point (*c*) is addressed by developing a simple parameterization based on *AX*_8_ sub-polyhedra that correlates with the tilt classification system of Glazer (1972 ▸). Details of these methodological improvements are given in the following section.

## 2. Elements of the revised crystal-chemical method

### 2.1. Pseudocubic representations of octahedra

**Figure 3:**
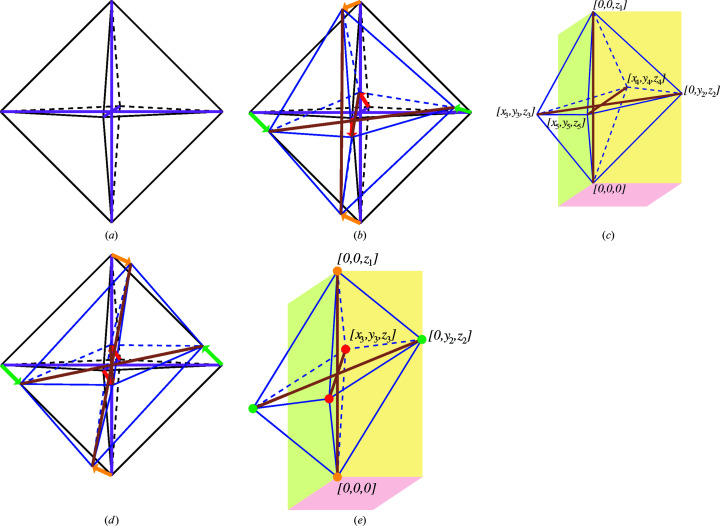
The number of independent parameters of a generalized, isolated centrosymmetric octahedron is equal to six. Views (*a*) to (*e*) discussed in §2.1.

In order that an object can be described as an octahedron, it requires three recognisable pairs of opposite vertices that may be linked to each another by vectors. If the ends of the stalks of a regular octahedron [Fig. 3 ▸(*a*)] are randomly displaced by limited amounts [Fig. 3 ▸(*b*)], the resulting form is still recognisable as an octahedron [Fig. 3 ▸(*c*)]. The six independent vertices lead to six sets of 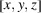 triplets in Cartesian space, *i.e.* 18 parameters. If only the form of the octahedron is relevant, and not its absolute position or orientation, these 18 parameters are reduced to 12 by subtracting six parameters: three to fix one vertex in space at [0,0,0] and three to define the orientation of the octahedron. In Fig. 3 ▸(*c*), one vertex has been fixed at [0,0,0] and the octahedron so rotated that the opposite vertex is fixed at [0 ,0, *z*_1_]. A further rotation of the octahedron about the *z* axis has been carried out to fix a vertex at [0, *y*_2_, *z*_2_]. The total of six coordinate-components equal to zero here signifies a reduction in independent parameters from 18 to 12, *i.e.* (*z*_1_; *y*_2_ *z*_2_; *x*_*n*_ *y*_*n*_ *z*_*n*_; *n* = 3,5). If, however, the translations at opposite ends of the three stalks are equal and opposite [Fig. 3 ▸(*d*)], a centrosymmetric octahedron results [Fig. 3 ▸(*e*)], in which the three stalks bis­ect one other at the centre of symmetry. Only one end of a stalk needs to be fixed in space in order to define the position of the opposite end, so that the number of independent parameters is halved to six [Fig. 3 ▸(*e*)]: 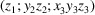 . This situation applies to all perovskites with *B* ions located at centres of symmetry.

**Figure 4:**
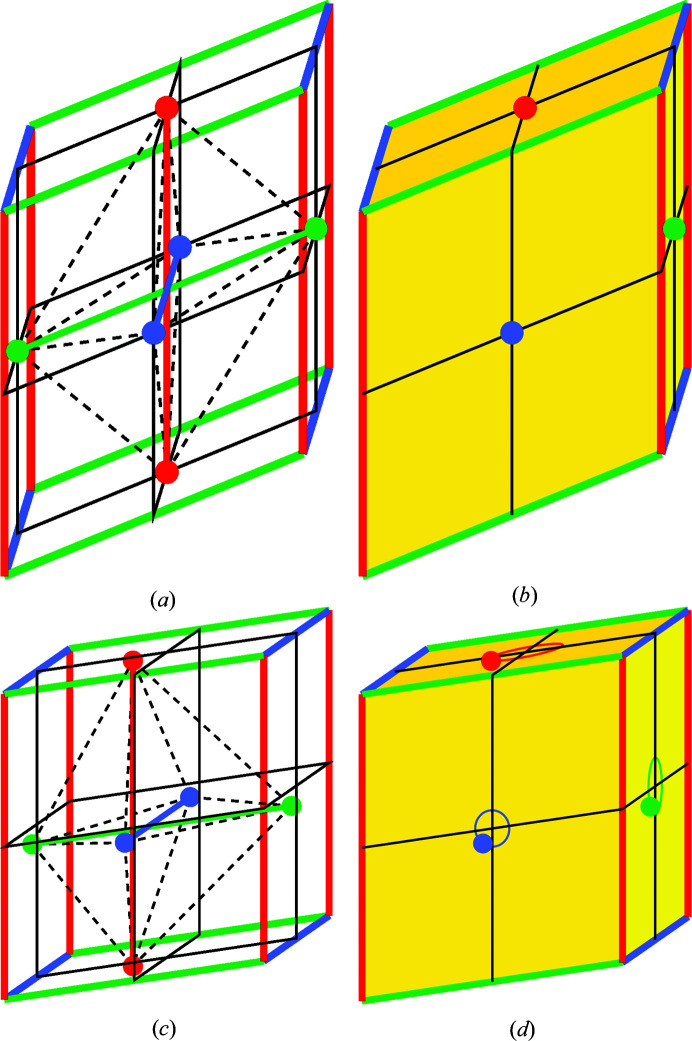
Visualization of the distortion of octahedra by enclosure in a pseudocube: (*a*), (*b*): centrosymmetric; (*c*), (*d*) non-centrosymmetric.

The six independent parameters of a centrosymmetric octahedron may be assigned to three stalk lengths 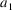 , 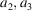 and three angles of intersection of the stalks, θ_12_, θ_23_ and θ_31_. In order to visualize the extent of its distortion, a parallelepiped enclosing the octahedron may be constructed by displacing the three stalk vectors to a common origin (Fig. 4 ▸).

As the three independent edge lengths of the parallelipiped are approximately equal, it may also be termed a pseudocube. Such a pseudocube is defined by the same six parameters as the octahedron, *i.e.* 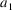 , 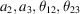 and 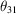 . It follows that regular octahedra would lead to pseudocubic representations of octahedra (PCRO) of cubic form.

In the case of the non-centrosymmetric octahedron of Fig. 3 ▸(*c*), the six octahedral vertices do not touch their parallelogram faces at the meeting points of the four quadrants [Fig. 4 ▸(*d*)]. The 12 independent parameters may be accommodated by noting the 2D polar coordinates 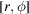 of the three emergent octahedral vertices, giving rise to additional parameters 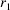 , 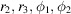 and 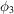 . Thus the PCRO construction is of general validity for visualizing distorted octahedra. Its parameterization may prove to be useful for characterizing non-centrosymmetric and polar perovskites in future work. The transformation between octahedron and PCRO is also reversible.

An advantage of the PCRO visualization method is that the aggregate distortion parameters representing normal and shear distortion developed for pseudocubic representations of tetrahedra (PCRT), 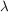 and 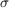 , (Reifenberg & Thomas, 2018 ▸; Fricke & Thomas, 2021 ▸) may be taken over without modification [equations (2), (3)].

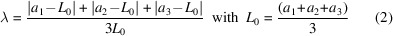




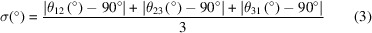

In this connection, the following values are obtained for the centrosymmetric and non-centrosymmetric octahedra of Figs. 3 ▸(*e*) and 3 ▸(*c*), respectively: [λ,σ] = [0.0367, 11.90°]; [λ,σ] = [0.0370, 7.10°]. It follows that the centrosymmetric octahedron displays a higher degree of angular distortion here. Furthermore, if the angular distortion parameters are expressed in radians instead of degrees, the relative degree of shear versus normal distortion can be quantified. Since 11.90° correspond to 0.21 and 7.10° to 0.12 radian, it follows that the shear distortion is greater than the normal distortion in both cases.

### 2.2. Parameterization of PCRO in terms of three vectors

The octahedra in perovskites are not isolated but form a three-dimensional corner-sharing network. Their crystal structural parameters, *i.e.* space group, unit-cell parameters and atomic coordinates, deliver full information on octahedral distortion, tilting and connectivity. Importantly, space group symmetry ensures connectivity. Once the distortion and tilting of a single octahedron in a structure with only one symmetry-independent octahedron has been defined, the distortions and tilting of all the other octahedra in the unit cell follow. To convey full information on the tilting of this single octahedron in a structure, its PCRO may be described by the three Cartesian stalk vectors, **a**_1_, **a**_2_ and **a**_3_ along its pseudocube edges. Generalized, analytical expressions for these vectors in different space groups may be derived inductively from known crystal structures. This process is described in §2.2.1 for space group *Pbnm* with *B* ions located at 4*b* special positions. The three vectors defining the axes of the pseudocubic unit cell are likewise defined in this Cartesian space, so that inclination angles of the stalks to the three pseudocubic axes, θ_1_, θ_2_ and θ_3_ may straightforwardly be calculated [see Fig. 1 ▸, equation (1) and §2.3]. In addition, the method also allows calculation of tilt angles ϕ_a_, ϕ_b_ and ϕ_c_ of the octahedra around the three pseudocubic axes, even when the octahedra are distorted.

#### 2.2.1. Space group *Pbnm* with *B* ions at 4*b* positions

The octahedral cage coordinating the *B* ion at 0½0 in the unit cell of orthorhombic space group *Pbnm* may be taken, this being the **cab** setting of space group 62 with standard symbol *Pnma*. 0½0 is one of the 4*b* special positions, with *X*1 ions in 4*c* special positions and *X*2 ions in 8*d* general positions (see Table S1 in the supporting information).^1^

Derivation of the analytical form of stalk, or equivalently PCRO vectors **a**_1_, **a**_2_ and **a**_3_ starts by taking an example structure, such as CaTiO_3_ at 296 K [Yashima & Ali (2009 ▸); ICSD code 162908]: *a* = 5.3709 Å, *b* = 5.4280 Å, *c* = 7.6268 Å; *x*(O1) = 0.0708, *y*(O1) = 0.4830, *x*(O2) = 0.7113, *y*(O2) = 0.2891, *z*(O2) = 0.0375; *z*(O1) has the fixed value of ¼.

The essential step is to convert from numerical fractional coordinates of the titanium ion and its six oxygen neighbours (Table 1 ▸, columns 2–4) to analytical fractional coordinates (Table 1 ▸, columns 8–10). One way to generate the numerical fractional coordinates would be to use a simple computer program to generate the Cartesian coordinates of the titanium and oxygen ions. (Table 1 ▸, columns 5–7). The numerical fractional coordinates (Table 1 ▸, columns 2–4) would then follow by multiplying these by the inverse orthogonalization matrix, in this trivial case 

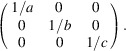

Letters a to f of the oxygen ions correspond to the atom labels in Fig. 1 ▸(*a*). They are based on the principle that the octahedral stalk from a to b is oriented closest to the *x*_PC_ axis in the positive direction, with the stalks from c to d and from e to f oriented closest to the positive *y*_PC_ and *z*_PC_ directions, respectively.

The analytical expressions in columns 8 to 10 of Table 1 ▸ are obtained by inspection: the values in columns 2 to 4 are compared with the starting values of *x*(O1), *y*(O1), *x*(O2), *y*(O2) and *z*(O2). It is important to note that the results depend on the convention used for expressing these parameters. For example, the alternative of *x*(O1) = 0.4292 and *y*(O1) = −0.0170, although symmetrically equivalent, would lead instead to the results *x*(O e) = *x*(O1) − ½ and *y*(O e) = −*y*(O1) + ½.

The three vectors for the PCRO are now formed in Table 2 ▸ by taking the differences in the analytical fractional coordinates for atom pairs (a,b), (c,d) and (e,f) given in Table 1 ▸. Cartesian vectors are formed by multiplying these differences by the orthogonalization matrix 

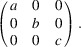

The Cartesian axes are parallel to the axes of the ortho­rhombic unit cell in this case.

By adopting this analytical representation, vectors **a**_1_, **a**_2_ and **a**_3_ are no longer tied to the example structure of CaTiO_3_: they apply to all perovskites in space group *Pbnm*, provided that the *B* ions occupy 4*b* sites and that the correct convention in choosing the values parameters *x*(O1), *y*(O1), *x*(O2), *y*(O2) and *z*(O2) has been followed. All eight crystallographic parameters are involved in defining the geometry of the PCRO in space group *Pbnm*: *a*, *b*, *c*, *x*(O1), *y*(O1), *x*(O2), *y*(O2), *z*(O2). For regular octahedra, six of these eight degrees of freedom (d.o.f.) would be used up in forming octahedra of a particular volume, with five defining the regular form and the sixth the volume. The remaining two d.o.f. would be used to define the unit-cell parameters and octahedral tilting. If the octahedra were only approximately regular, as is generally the case, more d.o.f. would be available for optimizing the octahedral tilting and unit-cell volume, in response to different *A* and *B* ion radii or to changing temperature or hydro­static pressure. Thus the idea of structural compromise in forming connected octahedral networks in perovskites can be modelled in response to varying (*p–T–X*) conditions.

#### 2.2.2. Other space groups

The other space groups analysed in this work are those that commonly arise in experimentally determined crystal structures. They span the following space groups: *Pbmn* (*B* ions in 4*a* sites), *Cmcm*, *Ibmm*, *P*4/*mbm*, *P*4_2_/*nmc*, *I*4/*mcm*, 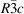 , 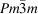 . Tables similar to Table 2 ▸ are generated for them in §S2 of the supporting information.

### 2.3. Implementation of the crystallographic to structural transformation in the Microsoft *Excel* Solver environment

Structural *analysis* requires a one-way transformation from crystallographic to structural parameters. For example, crystallographic parameters *a*, *b*, *c*, *x*_*i*_, *y*_*i*_, *z*_*i*_ are transformed to structural parameters such as PCRO parameters and tilt angles. By comparison, structural prediction requires a reversible transformation between the two parameter sets. The modeller will seek to establish systematic variations in the structural parameters with (*p–T–X*). If successful, interpolations and extrapolations to other (*p–T–X*) values can be made before reverse-transforming to crystallographic parameters. This technique has been demonstrated for olivines (Thomas, 2017 ▸), coesite (Reifenberg & Thomas, 2018 ▸) and quartz (Fricke & Thomas, 2021 ▸).

**Figure 5:**
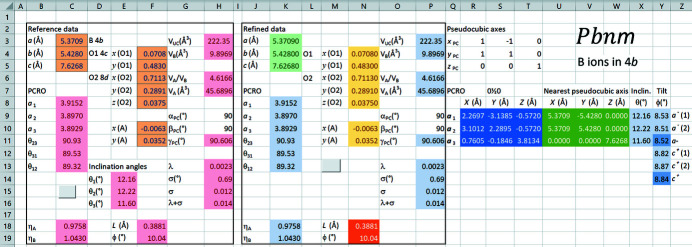
Screenshot of the *Pbnm* worksheet, which contains the reference data for CaTiO_3_ at 296 K (Yashima & Ali, 2009 ▸). No refinement has taken place, since the entries in the ‘Refined data’ and ‘Reference data’ boxes are identical.

The requirements of ease and flexibility of use together with a reversible transformation suggest that the Microsoft *Excel* Solver environment is appropriate for applying the method. In this connection, an *Excel* datafile already programmed is provided in the supporting information. This consists of eight worksheets for the different space groups. A screenshot of the worksheet for *Pbnm* with *B* ions in 4*b* positions is given in Fig. 5 ▸.

The user enters the crystallographic data in the ‘Refined data’ box in blocks K3:K5 (light-green background), N4:N8 and N10:N11 (mustard background), whereupon all the structural parameters are automatically calculated by *Excel*. The computational core of the spreadsheet is in blocks R9:T11 (deep-blue background) and U9:W11 (dark-green background). The formulas in these cells correspond to the entries in Table 2 ▸ and the pseudocubic axes in Cartesian coordinates, respectively. Values of all the structural parameters relating to octahedral distortion and tilting (light-blue background) can be seen as ways of describing the numerical values in these two blocks in a structurally meaningful way.

The purpose of the ‘Reference data’ box is threefold. First, it acts as a repository for the source, reference data (cells with orange background). Secondly, the dependent structural parameters can be calculated for this reference data by clicking on the button in cell C15.^2^ Thirdly, both reference data and dependent structural parameters can be used as constraints in structural refinements, which apply to the cells in the ‘Refined data’ box.

The different types of structural parameters, all with light-blue background, are summarized as follows.

a) **PCRO parameters** *a*_1_, *a*_2_, *a*_3_, θ_23_, θ_31_, θ_12_ are calculated as the lengths and intersectional angles of the vectors ***a***_1_, ***a***_2_, ***a***_3_ in block R9:T11. For example, 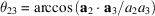 . **Aggregate distortion parameters** λ and σ [equations (2) and (3)] are calculated in block P13:P16, the latter quoted in both degrees and radians.

b) **Pseudocubic angle** γ_PC_ is calculated as the angle between pseudocubic axes *a*_PC_ and *b*_PC_ in cell P11: 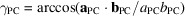 .

c) **Inclination angles** θ_1_, θ_2_, θ_3_ of the octahedral stalks are quoted in block X9:X11. These describe the relationship of the two sets of vectors in blocks R9:T11 and U9:W11 to one another. With respect to vector **a**_1_ in block R9:T9 and its nearest pseudocubic axis vector **a**_PC_ in block U9:W9, the following applies: 

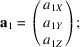




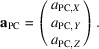

The subscripts 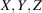 here denote the Cartesian 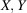 and *Z* components, respectively. It follows that

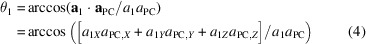

Substitution of 

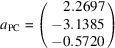

and 

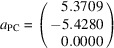

leads to θ_1_ = 12.16°. Angles θ_2_ and θ_3_ are derived from the corresponding pairs (**a**_2_,**b**_PC_) as well as (**a**_3_,**c**_PC_), respectively.

**Figure 6:**
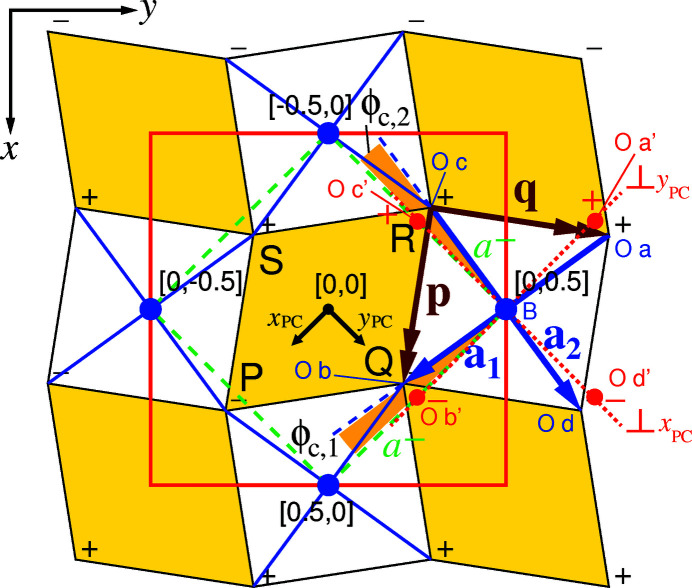
Tilted octahedra and basal parallelogram planes, *e.g.* PQRS, of *AX*_12_ polyhedra (in yellow) viewed along the orthorhombic *z* axis in space group *Pbnm*. The + and – signs in black denote *z* heights of the octahedral vertices relative to the *z* = 0 plane of the diagram. These are due to 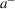 tilting. Green dashed lines show the directions of pseudocubic axes *x*_PC_ and *y*_PC_, the reference directions for the 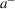 tilting. Red dotted lines represent the perpendicular planes to the green dashed lines, which are projected as lines in the *xy* plane. Red circles lie above the red dotted lines at the same *z* height as the octahedral vertices O a to O d. They are denoted by O a′ to O d′ and result from the four projections (O a → O a′), (O b → O b′), (O c → O c′) and (O d → O d′). 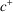 tilting causes the octahedra to be rotated about the orthorhombic *z* axis (or equivalently, the *z*_PC_ axis). The relevant tilt angles, ϕ_c,1_ and ϕ_c,2_, are shaded orange. (Vectors **p** and **q** are defined in §2.6.)

d) The method of calculation of angles of tilt ϕ_a_ and ϕ_c_ in the 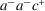 tilt system of space group *Pbnm* is shown in Fig. 6 ▸. These angles constitute an alternative to the inclination angles for relating the two sets of vectors in blocks R9:T11 and U9:W11 to each other. Since tilt angles are to be calculated for distorted octahedra, the method evaluates the projections of oxygen atoms O a, O b, O c and O d in the planes perpendicular to pseudocubic axes **a**_PC_, **b**_PC_ and **c**_PC_. These four atoms also define PCRO vectors **a**_1_ and **a**_2_: vector **a**_3_ does not influence the calculated tilt angles.

A tilt of type 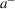 around **a**_PC_ is the angle between vector (O c′-O d′) and its projection in the *xy* plane. Similarly, an 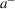 tilt around **b**_PC_ is the angle between vector (O a′-O b′) and its projection in the *xy* plane. Calculated values are shown in cells Y9 and Y10 in Fig. 5 ▸, with the mean value in cell Y11 (8.53°, 8.51° and 8.52°, respectively). The observed difference of 0.02° in calculated values results from an interplay of the deviation of γ_PC_ from 90° and the octahedral distortions.

The in-phase 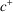 tilting causes the projections of the **a**_1_ and **a**_2_ octahedral vectors in the *xy* plane to be rotated away from pseudocubic axes *x*_PC_ and *y*_PC_ (Fig. 6 ▸). The two resulting tilt angles are given in cells Y12 and Y13 (8.82° and 8.87°), with the mean value in Y14 (8.84°).

The algorithms for calculating these tilt angles are described in §S3 of the supporting information, where reference is also made to their implementation in the *Excel* file in the supporting information.

e) Unit-cell volume *V*_UC_, polyhedral volumes *V_B_*, *V_A_* and volume ratio *V*_*A*_/*V*_*B*_ are calculated in blocks P3:P4 and P6:P7. *V*_UC_ = 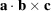 and 

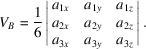

The latter calculation method is facilitated by the centrosymmetry of the octahedron. Volume ratio *V_A_/V_B_* follows from these parameters as [(*V*_UC_/*Z*) − *V*_*B*_]/*V*_*B*_ and 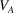 as 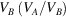 .

f) **Parameters η_*A*_ and η_*B*_** are likewise extracted from the core computational data (blocks R9:T11 and U9:W11) and defined in §2.6.

g) **Parameters *L* and ϕ** (block N18:19 with brown background) relate to the *A* ion positions and are independent of the anionic network. They are defined in §2.7.

### 2.4. Example Solver refinements

Initial insight into use of the Solver is gained here by addressing an issue relevant to the historical development of perovskite structural chemistry: the ability of different space groups to accommodate regular octahedra. A comparison is also made between tilt angles calculated as in point d) of §2.3 and the values yielded by commonly used approximations.

**Figure 7:**
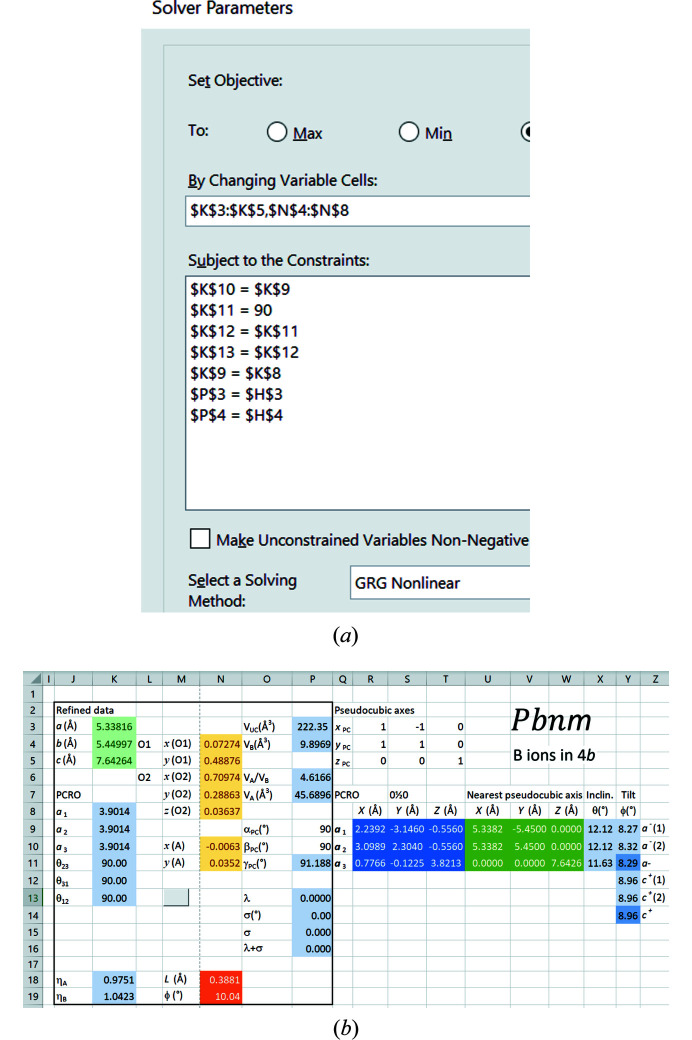
(*a*) Solver settings for generating regular octahedra in space group *Pbnm* with unchanged unit cell and octahedral volumes. (*b*) Results of the refinement.

The five degrees of freedom required to define the regular octahedral form (see §2.2.1) result in the constraints a) *a*_2_ = *a*_1_; b) *a*_3_ = *a*_2_; c) θ_23_ = 90°; d) θ_31_ = θ_23_; and e) θ_12_ = θ_31_. Since there are eight d.o.f. in total, two further constraints may be applied in the refinement. For example, it may be stipulated that the unit cell and the octahedral volumes remain unchanged: f) *V*_UC_ = *V*_UC,reference_ and g) *V*_*B*_ = *V*_*B*,reference_. The coding of these constraints in the Solver in shown in Fig. 7 ▸(*a*), whereby constraints f) and g) also refer to cells $H$3 and $H$4 in Fig. 5 ▸. The end-point of this tightly defined refinement is shown in Fig. 7 ▸(*b*): a cubic PCRO of side length 3.9014 Å is generated, corresponding to a regular octahedron with perpendicular stalks. The values of the other structural parameters described in §2.3 appear in the cells with a light-blue background.

The crystal structures obtained by applying the same refinement conditions to reference structures in all the space groups are given in Table 3 ▸. The Solver constraints for the respective space groups are pre-programmed in the *Excel* file in the supporting information.

For space group *P*4_2_/*nmc*, the obtaining of regular octahedra in tilt system *a* ^+^ *a* ^+^ *c* ^−^ is at variance with the conclusion of Howard & Stokes (1998 ▸). A resolution of this discrepancy is thought likely to depend on the observed interdependence of the coordinates of atoms O2 and O3 in the refined structure (Table 3 ▸), which implies a refinement into higher symmetry or pseudo-symmetry than *P*4_2_/*nmc*.

The values of tilt angles below the dividing line in Table 3 ▸ serve to compare the values calculated by the method given in §2.3 and approximations for tilt angles used by other authors. Following an initial analytical treatment of perovskite tilt angles by Megaw (1973 ▸), a more developed treatment according to space group was given by Kennedy, Prodjosantoso *et al.* (1999 ▸) as follows. The four parameterizations a) to d) here correspond to headers a) to d) in the row ‘Method’ in Table 3 ▸.

a) *Pbnm* (*B* ions in 4*a*): O2: 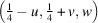 . 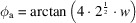 ; 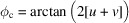 .

b) *Cmcm*: O1: 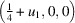 ; O2: 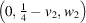 ; O3: 
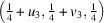
 .


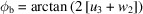
 ; 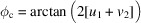 .

c) *I*4/*mcm*: O: 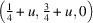 . 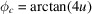 .

Mountstevens *et al.* (2003 ▸) augmented this set with a result for space group *Imma*, which upon resetting in *Ibmm*, is as follows:

d) *Ibmm*: O2: 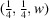 . 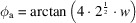 .

This expression is equivalent to *Pbnm* with *u* = *v* = 0.

In general, the agreement between values generated in this work and approximations a) to d) above is satisfactory for the in-phase tilts, ϕ_c_. Antiphase angles ϕ_a_ for space groups *Pbnm* and *Ibmm* show poorer agreement. In order to investigate whether this is a systematic discrepancy, the structural data of Kennedy, Prodjosantoso *et al.* (1999 ▸) for CaTiO_3_ (*Pbnm*) at 1273 K were fed into the *Excel* program, yielding the following two sets of tilt values and inclination angles θ_3_: ϕ_a_ = 6.53°; ϕ_a,refined_ = 6.59°; θ_3_ = 8.92°; θ_3,refined_ = 9.24°. These are to be compared with the tilt values given by the Kennedy approximation: ϕ_a_ = 9.19°; ϕ_a,refined_ = 9.24°. These results and those in the *Pbnm* (B: 4*a*) and *Ibmm* columns of Table 3 ▸ lead to the following two conclusions: (1) The ϕ_a_ angles calculated by the method of §2.3 are systematically lower than those generated by the Kennedy approximation; (2) The Kennedy approximation generates exact values of inclination angle for refined structures with regular octahedra, *i.e.* θ_3,refined_, and not tilt angle ϕ_a_ as defined in this work.

In the case of *Cmcm*, good agreement for ϕ_a_ between §2.3 and Kennedy, Prodjosantoso *et al.* (1999 ▸) is obtained for regular octahedra in the refined structure, but not for the distorted octahedra in the unrefined structure.

A rationalization for the smaller values of ϕ_a_ generated by the method of §2.3 is that the inclination angle θ_3_ of a regular octahedron will always be larger than an angle within a plane of projection perpendicular to a pseudocubic axis (Fig. 6 ▸). The former angle is generated by the approximations of Kennedy, Prodjosantoso *et al.* (1999 ▸) and the latter angle by the method of §2.3.

### 2.5. PCRO parameters, inclination angles and tilt systems with numbers of degrees-of-freedom by space group

Whether regular octahedra can be formed in a particular space group depends on whether the octahedra have sufficient degrees of freedom (d.o.f.). The number of d.o.f. defining the octahedral anionic network is *N*(UC) + *N*(*X*), of which *N*(tilt) are used to define independent tilt angles. In *P*4_2_/*nmc* with irregular octahedra, the tilt system is 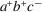 , *i.e.* *N*(tilt) = 3. For regular octahedra, this is reduced to 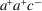 with *N*(tilt) = 2. The parameter denoting the remaining d.o.f. available to construct the octahedra, *N*(PCRO), is equal to *N*(UC) + *N*(*X*) − *N*(tilt) (Table 4 ▸). In space groups *Pbnm* and *Cmcm*, *N*(PCRO) values equal to six signify a capacity to form regular octahedra independently of the values of the two tilt angles. Although *N*(PCRO) is less than 6 in *Ibmm*, *I*4/*mcm*, *P*4/*mbm* and 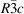 , this does not preclude the formation of regular octahedra, since the space group symmetry itself provides partial regularity: in *Ibmm*: *a*_1_ = *a*_2_ and θ_23_ = θ_31_; in *I*4/*mcm* and *P*4/*mbm*: *a*_1_ = *a*_2_ with θ_23_ = θ_31_ = θ_12_ = 90°; in 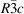 : *a*_1_ = *a*_2_ = *a*_3_ with θ_23_ = θ_31_ = θ_12_. In space group *P*4_2_/*nmc*, the *N*(PCRO) value of less than 6 signifies that the two tilt angles in the refined structure will be interdependent.

The final two rows in Table 4 ▸ relate to the d.o.f. assigned to *A* and *B* cations. The structures associated with the *A* cations are discussed in §2.7, after the primary structures associated with the parameter *N*(tilt) have been considered in the following sub-section.

### 2.6. Additional anionic network parameters related to octahedral tilting

Since octahedral tilting *per se* is a secondary structural feature relative to the *BX*_6_ octahedra, the primary structures resulting from this tilting necessarily concern the coordination of the *A* ions. It has long been customary to assume *AX*_8_ polyhedra in GdFeO_3_-type perovskites such as CaTiO_3_ in space group *Pbnm* (Liu & Liebermann, 1993 ▸). Mitchell & Liferovich (2004 ▸), adopting the perspective of coordination chemistry, reported an increase in coordination number from 8 to 9 with *x* in the solid solution series Ca_1–*x*_Na_*x*_Ti_1–*x*_Ta_*x*_O_3_. Mitchell (2002*b* ▸) states generally that coordination numbers of 8, 9, 10 and 12 are possible, and calculates as an example the volume of uncoordinated space in SrZrO_3_ (*I*4/*mcm*) as 8.7%. This is the difference between *V_A_*, the volume assigned to *AX*_12_ polyhedra from volume filling with *BX*_6_ octahedra, and the volume of a more appropriate *AX*_8_ polyhedron, 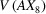 .

**Figure 8:**
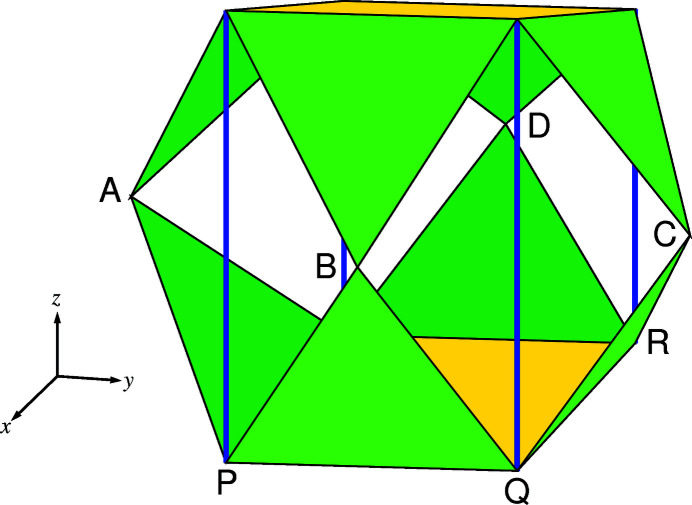
*AX*_8_ and *AX*_12_ polyhedra in space group *Pbnm*. The two opposite, yellow parallelogram faces are joined by vertical blue struts to form *AX*_8_ polyhedra. The eight green faces are shared with *BX*_6_ octahedra.

From a functional viewpoint, the *AX*_8_ polyhedra in tilted perovskites provide scaffolding to support the eight octahedral faces of the full *AX*_12_ polyhedra. In *Pbnm*, the inner *AX*_8_ polyhedron is formed by the top and bottom parallelogram *AX*_12_ faces and four vertical struts (Fig. 8 ▸).

It is now appropriate to interpret Fig. 6 ▸ in terms of the effect of octahedral tilting on *A*-ion coordination. The *c* ^+^ tilting causes parallelograms PQRS to be formed instead of rectangles, with a concomitant reduction in area *A*(*PQRS*). The *a^−^* tilting, by comparison, leads to equal and opposite displacements of vertices *P*, *Q*, *R* and *S* along *z* (denoted by + and −). The volume contributions made by plane PQRS and the other five faces of the *AX*_8_ polyhedron to 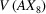 are not affected by this tilting, since changes brought about by the equal and opposite displacements relative to the centre-of-symmetry at [0,0,0] cancel one another out.^3^ The mirror plane through A, B, C and D in all space groups with *c* ^+^ tilting means that the horizontal positions, in this case the *x* and *y* coordinates of the bottom and top parallelograms in Fig. 8 ▸, are identical. It follows that the formula *V*(*A* *X*_8_) = *A*(*PQRS*)(*c*/2) could be used, since the centres of both parallelograms are separated by *c*/2 in all space groups with in-phase *c*-axis tilting apart from *P*4/*mbm*, where the separation is *c*. In space groups *P*4_2_/*nmc* and *I*4/*mcm*, to which anti-phase, *c* ^−^ tilting applies, the same formula is valid, since the mean cross-section 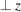 is 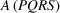 .

Since the 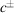 component of the tilting is solely responsible for the reduction in *A*(*PQRS*) and *V*(*A* *X*_8_), the converse applies that *A*(*PQRS*) quantifies the extent of this tilting. Without 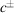 tilting, *A*(*PQRS*) would be equal to one quarter of the unit cell cross-sectional area, *i.e.* (*ab*)/4 in crystal systems with perpendicular *x* and *y* axes. A dimensionless coefficient 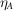 to quantify the amount of 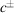 tilting may therefore be defined as follows.

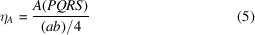




Although the 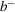 tilting about the *x*_PC_ and *y*_PC_ axes in *Pbnm*, more generally the 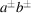 tilting component when applied to all space groups, does not affect 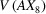 , it does affect 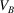 . For a given perovskite compound with unit-cell cross-section *ab*, this tilting allows larger 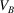 values than would otherwise have been the case. The greater the degree of tilting, the larger the 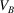 value. A dimensionless coefficient 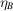 to quantify this is given by relating 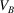 to the volume of an upright, untilted octahedron of equal basal area in *xy* projection, 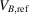 . The in-plane components of vectors **p** and **q** in Fig. 6 ▸ define the waist of this octahedron of perpendicular height *c*/2. These depend in turn on PCRO vectors **a**_1_ and **a**_2_.

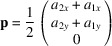

 and 

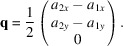




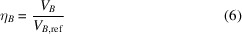




In the presence of 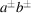 tilting, η_*B*_ > 1. *V_B_* is calculated as one-sixth of the volume of the PCRO and *V*_*B*,ref_ as twice the volume of a right pyramid of height 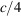 with parallelogram base of area 

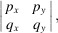


*i.e*.

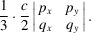

Analytical expressions for 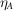 and 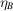 are quoted in Table 5 ▸. The form of 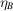 is similar for all space groups, and 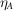 can generally be traced back to simple expressions involving fractional coordinates. Particularly significant are the fixed, limiting values of 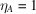 and 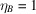 in space groups *Ibmm* and *I*4/*mcm*, respectively. These govern the sequence of phase transitions in series with increasing *V_A_* volume (see §3.1). The corresponding derivations are in §S4 of the supporting information, along with diagrams of the 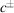 and 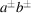 tilt patterns determining 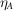 and 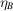 , respectively. These parameters are also calculated in the *Excel* datafile in the supporting information.

Since 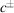 tilting reduces 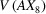 and therefore 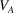 , whereas 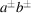 tilting increases 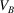 , use of *V_A_/V_B_* as an indicator of the overall degree of tilting is vindicated. By using parameters 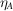 and 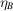 to quantify the tilting, which are simply calculated, the difficulties of defining and calculating tilt angles unequivocally can be circumvented. As may be inferred from the complexity of Fig. 6 ▸ and the comparison of calculated tilt angles in the lower part of Table 3 ▸, this could be seen as an advantage.

### 2.7. *A* cation positions

In earlier work on the parameterization of centrosymmetric perovskites (Thomas, 1998 ▸), difficulties were experienced in fixing the positions of the *A* ions by reference to their coordinating *X* ions in the *AX*_12_ polyhedra. However, subsequent work by Magyari-Köpe *et al.* (2001 ▸, 2002 ▸) demonstrated that their positions could be treated as a function of the *V_A_/V_B_* ratio.

**Figure 9:**
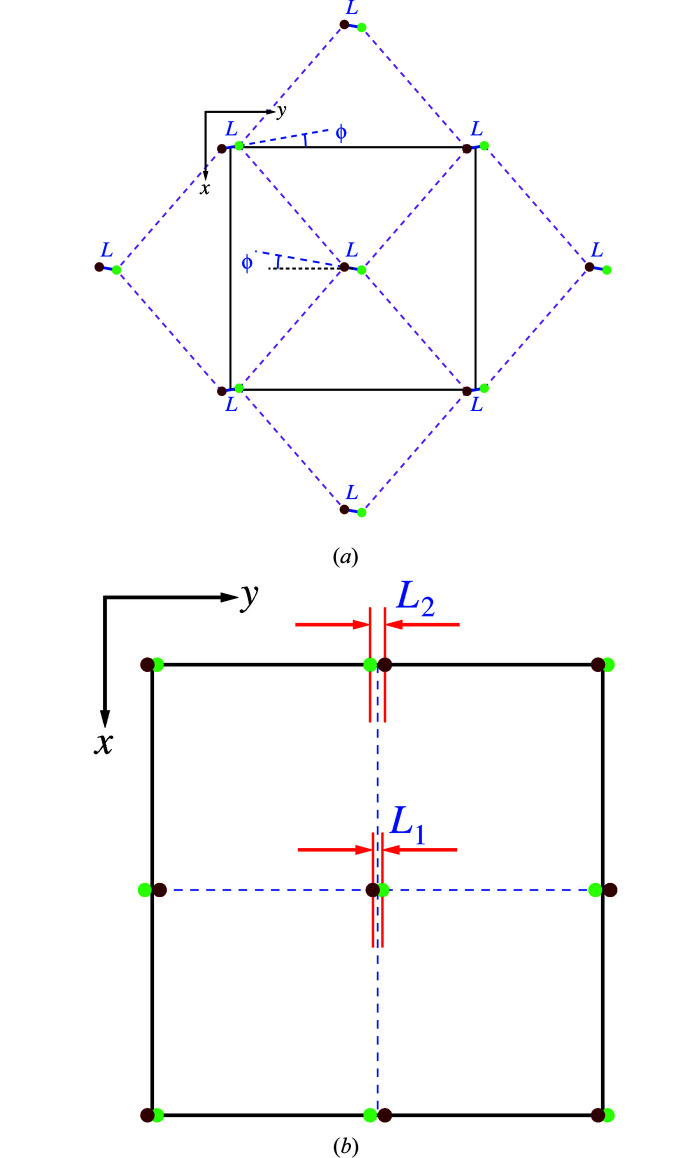
*A* cation positions in space groups (*a*) *Pbnm* and (*b*) *Cmcm* in 2D projection. Green circles: height +¼; brown circles: height −¼. A network with dashed lines is shown in (*a*).

The essential objective is to quantify the small displacements from geometrically regular positions [0, 0] and [½, ½] in the relevant plane of projection (Fig. 9 ▸). The parameterization applied here is limited to a transformation to polar coordinates *L* and ϕ in *Pbnm* [Fig. 9 ▸(*a*)], whereby *L* is twice the displacement of an individual ion. In space group *Cmcm*, two independent lengths, *L*_1_ and *L*_2_, apply [Fig. 9 ▸(*b*)]. These displacements are either along one axis or non-existent in the other space groups.

## 3. Analysis of structures at variable temperature and chemical composition

The structural parameters defined in §2 are used here to characterize the sequences of phase transitions observed with increasing temperature and varying chemical composition in centrosymmetric perovskites. Li *et al.* (2004 ▸) identified the following commonly occurring sequence with increasing temperature or mean *A* ion radius: *Pbnm* → *Ibmm* → *I*4/*mcm* → 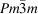 . This applies to Sr_1−*x*_Ba*_x_* HfO_3_ and Sr_1–*x*_Ba*_x_* ZrO_3_ perovskites (Kennedy *et al.*, 2001 ▸), as well as to the systems Sr_1–*x*_Ba*_x_* SnO_3_ and Ca_1–*x*_Sr*_x_* SnO_3_ (Mountstevens *et al.*, 2003 ▸). Identification of the intermediate *Ibmm* phase was significant, as it had previously been regarded as rare (Kennedy *et al.*, 2001 ▸). The temperature range of stabilization of this phase is highly variable, with values greater than 570 K in BaPbO_3_ (Fu *et al.*, 2007 ▸), of 140 K in SrRuO_3_ (Kennedy *et al.*, 2002 ▸), 90 K in SrHfO_3_ (Li *et al.*, 2004 ▸) and less than 20 K in SrRhO_3_ (Kennedy *et al.*, 2004 ▸). It is characterized by a fixed value of parameter 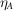 equal to one (Table 5 ▸).

A second sequence was identified by Ahtee & Darlington (1980 ▸) for NaTaO_3_: 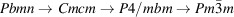 , with further structural work by Kennedy, Prodjosantoso *et al.* (1999 ▸) reporting transition temperature ranges of 738–753 K, 823–863 K and 883–913 K. More recently, Mitchell *et al.* (2014 ▸) reported the same series of phase symmetries for the mineral lueshite, NaNbO_3_.

Both sequences are characterized by an increasing *V_A_/V_B_* ratio with *T* or *x*, which may be attributed either to a larger volume expansion coefficient of *V_A_* relative to *V_B_* or to a compositionally induced expansion of the *A*-site volume relative to the *B* site. The term ‘*A*-ion perturbation’ may be applied in both cases, since a change in the *A*-site geometry is the dominant driving force causing the whole structure to respond.

A solid solution series with a rising *V_A_/V_B_* ratio brought about by reducing *V_B_* has been established by Yang (2008 ▸) for LaCr_1–*x*_Ni*_x_* O_3_ at room temperature. Upon increasing *x* from 0 to 0.7, a *Pbnm* → 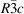 phase transition is observed. In this case, a *B*-ion perturbation is being applied.

### 3.1. *x*- and *T*-series with *A*-ion perturbations

#### 3.1.1. Sr*_x_* Ba_1–*x*_SnO_3_ and Sr*_x_* Ba_1–*x*_HfO_3_

Table 6 ▸ contains structural parameters calculated from the refinements of Mountstevens *et al.* (2003 ▸) for the solid solution series Sr*_x_* Ba_1–*x*_SnO_3_ and of Li *et al.* (2004 ▸) for the Sr*_x_* Ba_1–*x*_HfO_3_ series.

On reading Table 6 ▸ from top to bottom, values of *V_A_* confirm that the increasing mean *A*-ion radius is the principal driving force for the structural changes in both systems. *V_A_* rises by 7.2% between *x* = 0 and *x* = 1 in the tin-containing and by 8.4% in the hafnium-containing series. Furthermore, the larger ionic radius of Hf^4+^ compared to Sn^4+^ in sixfold coordination [0.71 *cf.* 0.69 Å; Shannon (1976 ▸)] causes the *V_A_* value at a given Sr:Ba ratio to be systematically larger in the system with hafnium as *B* ion. By comparison, the volume of the *B* site increases by 1.4% between *x* = 0 and *x* = 1 in the tin-containing series compared system compared to 2.3% in the hafnium series. The following ranges of the *V_A_*/*V_B_* parameter span the *Ibmm* phase in the two systems: 4.824 < *V_A_*/*V_B_* < 4.976 and 4.81 < *V_A_*/*V_B_* < 4.926.

**Figure 10:**
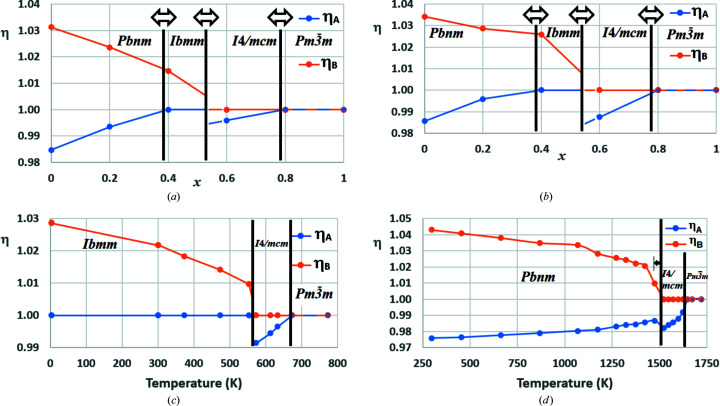
Characterization of phase transitions in terms of tilt-related parameters η_*A*_ and η_*B*_: (*a*) Sr*_x_* Ba_1–*x*_SnO_3_, (*b*) Sr*_x_* Ba_1–*x*_HfO_3_, (*c*) BaPbO_3_, (*d*) CaTiO_3_. Circles denote experimentally determined points, which are joined by straight lines. Arrows signify limited flexibility in the positions of the phase transitions.

The variation of parameters η_*A*_ and η_*B*_ with *x* reveals the mechanisms behind the *Pbnm* → *Ibmm* and *Ibmm* → *I*4/*mcm* phase transitions in these series [Figs. 10 ▸(*a*) and 10 ▸(*b*)].

Reading these two diagrams from left to right, the *Pbnm* → *Ibmm* transition is triggered by η_*A*_ reaching the limiting value of 1 whilst η_*B*_ is still > 1. The range of stability of the *Ibmm* phase is determined by the *x* value at which η_*B*_ becomes equal to 1, whereupon a phase transition to *I*4/*mcm* takes place. The diagrams suggest that the approach of η_*A*_ to 1 takes place continuously at the *Pbnm*–*Ibmm* boundary. By comparison, the *Ibmm*–*I*4/*mcm* boundary is characterized by a discontinuous step in η_*B*_ down to 1. Also characteristic of the latter boundary is a fall in η_*A*_ to a value less than 1. This may be understood by considering that η_*A*_ and η_*B*_ can only both be equal to one in the aristotype 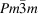 phase. However, at the start of the range of stability of the *I*4/*mcm* phase, *V_A_*/*V_B_* has not reached the limiting value of 5. The transition from *I*4/*mcm* to 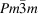 takes place as soon as *V_A_* has increased sufficiently for this *V_A_*/*V_B_* limit to be reached.

#### 3.1.2. BaPbO_3_

The above rationalization can be transferred without modification to series where temperature is responsible for an increasing *V_A_* volume. For example, the *Ibmm* phase is stabilized over a wide temperature range in BaPbO_3_ perovskites, these being of technological relevance due to superconductivity in the Ba-Pb-Bi-O system. Following earlier structural refinements by Moussa *et al.* (2001 ▸) and Ivanov *et al.* (2001 ▸) in the monoclinic space group *C*2/*m* on cooling and at room temperature, respectively, Fu *et al.* (2005 ▸) interpreted this monoclinic distortion as probably due to twinning. They also provided four structural refinements in *Ibmm* at room temperature and at 4.2 K. Upon heating, Fu *et al.* (2007 ▸) reported phase changes to tetragonal *I*4/*mcm* and thereafter to 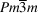 at approximate temperatures of 573 and 673 K respectively. Fig. 10 ▸(*c*) is generated from experimental points calculated from the structural refinements of Fu *et al.* (2005 ▸, 2007 ▸). The absence of *Pbnm* symmetry in BaPbO_3_ can be ascribed to the *V_A_*/*V_B_* ratio remaining above 4.8320 (8) over the temperature range from 4.2 K to 773 K, this being higher than the threshold values of 4.81 and 4.824 noted in §3.1.1 for *Ibmm* symmetry. This interpretation is supported by the stabilization of the strontium compound SrPbO_3_ in *Pbnm* (Fu & Ijdo, 1995 ▸). The calculated *V_A_*/*V_B_* value here is significantly lower, at 4.418 (2).

Despite the absence of a *Pbnm* phase in BaPbO_3_, Fig. 10 ▸(*c*) shows that the *Ibmm* → *I*4/*mcm* transition is also governed by discontinuous jumps in η_*B*_ to 1 and η_*A*_ to less than 1, as in Figs. 10 ▸(*a*) and 10 ▸(*b*). The approach to the aristotype phase follows the same principles as noted earlier.

#### 3.1.3. CaTiO_3_

The mineral perovskite, CaTiO_3_, has not been observed in space group *Ibmm*, although it exists in *Pbnm* at room temperature (Sasaki *et al.*, 1987 ▸) and at temperatures up to 1373 K (Liu & Liebermann, 1993 ▸). Redfern (1996 ▸) paved out the sequence of phase transitions at yet higher temperatures through *I4/mcm* to and 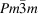 aristotype, reporting transition temperatures of 1373–1423 K and at ∼1523 K. Although Kennedy, Howard & Chakoumakos (1999 ▸) subsequently raised the possibility of an orthorhombic *Cmcm* structure being stabilized at around 1380 K, this was refuted by Ali & Yashima (2005 ▸), who later generated the nineteen structural refinements upon which the experimental points of Fig. 10 ▸(*d*) are based (Yashima & Ali, 2009 ▸). It is logical that no intermediate phase of symmetry *Ibmm* is formed with increasing temperature, since η_*A*_ remains significantly below one over the whole *Pbnm* range. This means that the degree of *A*O_8_ expansion induced thermally is smaller than the compositionally induced *A*O_8_-expansion analysed in §3.1.1. Stabilization of the *Pbnm* phase is curtailed by η_*B*_ falling to 1 at the *Pbnm*–*I*4/*mcm* boundary. After the temperature of this phase transition has been reached, the overall expansion of the unit cell allows the volumetric requirements of the TiO_6_ octahedron to be satisfied without 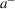 tilting in space group 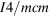 . The remaining tilting allows further comparatively greater *A*O_8_ expansion with increasing temperature up to the temperature of the phase transition to 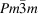 .

In all four series upon which Fig. 10 ▸ is based, the changes in tilt systems taking place at the phase transitions, these being critical events, are largely determined by the systematic changes in tilt angles taking place over wide compositional or temperature ranges. Parameters η_A_ and η_B_ are ideally suited for encapsulating these changes.

#### 3.1.4. NaTaO_3_ and NaNbO_3_

These compounds provide an experimental basis for assembling the factors governing stabilization of the *Cmcm* phase, and more widely, the progression from *Pbnm* to 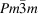 via *Cmcm* and *P*4/*mbm* instead of *Ibmm* and *I*4/*mcm*. *I*4/*mcm* and *P*4/*mbm* structures are very similar, the only difference being anti-phase or in-phase *c* axis tilting, respectively. Furthermore, structural parameters *V_A_*/*V_B_*, η_*A*_ and η_*B*_ cannot discriminate between these two structures. It is therefore appropriate to regard *Cmcm* and *Imma*, in the first instance, as alternative precursor phases to a generic phase of symmetry *I*4/*mcm* or *P*4/*mbm*.

An analysis of the phases of *Cmcm* symmetry in terms of *V_A_*/*V_B_*, η_A_ and η_B_ within these two compounds is given in Table 7 ▸.

The definitive characteristic of the *Cmcm* phase is the proximity of η_*B*_ values to one in Table 7 ▸. This parameter has much larger values in the *Ibmm* phase, with η_*B*_ values greater than 1.03 observed [see Table 6 ▸ and Figs. 10 ▸(*a*), 10 ▸(*b*) and 10 ▸(*c*)]. This difference is due to there being only one tilt of type 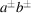 in *Cmcm*, compared to two in *Ibmm* (see §S4 of the supporting information). Values of η_*B*_ close to one confirm *Cmcm* as a precursor to an *I*4/*mcm* or *P*4/*mbm* phase, in which η_*B*_ is exactly equal to one. The existence of *Cmcm* symmetry in CaTiO_3_ at ∼1380 K, as proposed by Kennedy, Howard & Chakoumakos (1999 ▸), is indeed possible, since η_*B*_ is close to one at this temperature.

Also to be noted in Table 7 ▸ is the splitting of η_*A*_ values made possible by there being two symmetry-independent *A* sites in *Cmcm*. Geometry simply requires a mean value 〈η_*A*_〉 = (η_*A*1_ + η_*A*2_)/2 of less than one. This splitting is considerably more marked in NaNbO_3_. The ability for the lower η_*A*_ value to be significantly less than one could go some way towards rationalizing the phase coexistence of *Pbnm* and *Cmcm* phases at room temperature observed in NaTaO_3_ by Knight & Kennedy (2015 ▸). A further contributory factor towards stabilization of *Cmcm* in 1 mol% K-doped samples of NaTaO_3_ at room temperature (Arulnesan *et al.*, 2016 ▸) could be preferential occupation of the sites with higher η_*A*_ value by the larger potassium ions, *i.e.* a partial ordering.

It is speculated that the proximity of η_*B*_ to one in *Cmcm* is conducive to a to a higher temperature phase transition to *P*4/*mbm*, whereas the sudden fall in η_*B*_ within *Ibmm* or *Pbnm* phases observed in Figs. 10 ▸(*a*)–10 ▸(*d*) favours a transition to *I*4/*mcm*.

### 3.2. *X*-series with *B*-ion perturbations

Calculated structural parameters for the LaCr_1–*x*_Ni*_x_* O_3_ solid solution series (Yang, 2008 ▸) are given in Table 8 ▸.

Unlike the structures perturbed by *A* ions, the variation in η_*A*_ and η_*B*_ parameters is small and also unsystematic for structures in space group *Pbnm*. There is no tendency of either parameter to approach one. The expected cross-correlations between parameter pairs η_*A*_ ↔ ϕ_c_ and η_*B*_ ↔ ϕ_a_ are observed within the *Pbnm* phase field: the greater the magnitude of the deviations of η_*A*_ and η_*B*_ from one, the larger the 〈ϕ_c_〉 and 〈ϕ_a_〉 angles. The ϕ_a_ tilt angle falls to 4.64° and the mean tilt angle (〈ϕ_a_〉 + 〈ϕ_c_〉)/2 to 5.50° in *Pbnm* just before the phase transition, with ϕ_a_ rising to 5.97° in the 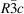 phase at *x* = 0.7. A gradual reduction in ϕ_a_ to 5.72° is observed up to *x* = 1, this still being a stable rhombohedral phase far from a transition to the cubic aristotype. The gradual fall in *V_B_* with *x* is consistent with the smaller ionic radius of Ni^3+^ compared to Cr^3+^ (Shannon, 1976 ▸) and is responsible for the systematic increase in *V_A_*/*V_B_* with *x*, which peaks at 4.841 in *Pbnm* before the phase transition to 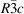 and falls to 4.809 thereafter. The 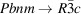 transition is examined further in §4.4.

## 4. Modelling the structural variation of *Pbmn* perovskites with increasing pressure

The structures adopted by perovskites under increasing hydro­static pressure rest on the different compressibilities of the *AX*_12_ and *BX*_6_ polyhedra (Angel *et al.*, 2005 ▸), such that the volume ratio *V_A_*/*V_B_* may either increase or decrease. In general, experimental difficulties limit the pressure range over which full structure refinements can be obtained, a common practice being to report the variation of unit-cell parameters over a wider pressure range by means of the third-order Birch–Murnaghan equation of state [equation (7)].

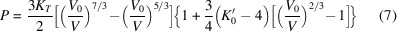

Here, coefficient 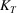 is the bulk modulus and 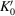 is its derivative with respect to pressure. 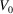 represents the reference unit-cell volume. The appropriate notation to use for the variation of cell parameters *a*, *b*, *c* with pressure would be 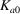 , 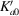 , 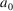 *etc*., whereby cubes 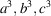 will have been used for the Birch–Murnaghan fitting. It is also common to quote linear compressibilities 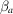 , 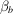 , 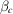 , whereby 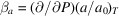 , *etc.*, these generally holding over a limited pressure range. By setting 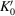 values equal to 4, the term in curly brackets is equal to one, giving rise to the second-order Birch–Murnaghan equation.

The primary motivation for studying perovskites under pressure has been to simulate MgSiO_3_ perovskite in the lower mantle, for which a phase transition to a post-perovskite phase at pressures > 125 GPa and a temperature of 2700 K has been proposed (Murakami *et al.*, 2004 ▸). This system is simulated by assuming regular octahedra in the following sub-section.

### 4.1. Simulation of MgSiO_3_ under pressures of up to 125 GPa

It may be assumed that MgSiO_3_ remains in space group *Pbnm* at all pressures up to ∼125 GPa. Accordingly, the 12 sets of *a*, *b*, *c* unit-cell parameters reported by Vanpeteghem *et al.* (2006 ▸) at pressures up to 10 GPa were used to derive the following Birch–Murnaghan constants to second order for the unit-cell volume and *a* and *c* unit-cell parameters^4^: 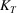 = 252.90 GPa; 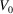 = 162.52 Å^3^; 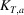 = 232.11 GPa; 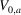 = 109.08 Å^3^; 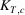 = 240.78 GPa; 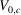 = 328.33 Å^3^. 

The assumption was made that these constants could extended to 125 GPa, thereby yielding theoretical *a*, *b* and *c* unit-cell parameters over this whole range. This process was validated by comparing the values obtained (‘Extrapolated  B-M’) with values quoted by Murakami *et al.* (2004 ▸) and Fiquet *et al.* (2000 ▸). Acceptable agreement is found in Table 9 ▸.

**Figure 11:**
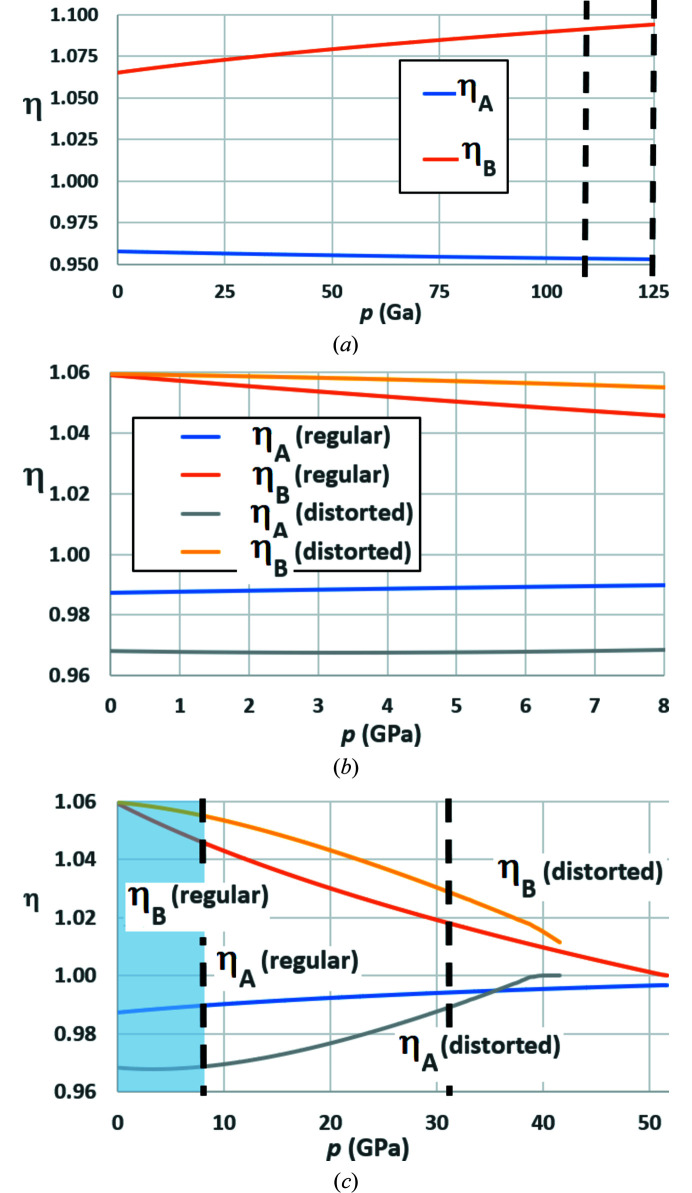
Simulations of MgSiO_3_ and YAlO_3_ in terms of η_*A*_ and η_*B*_. (*a*) MgSiO_3_ with regular octahedra up to *p* = 125 GPa; (*b*) comparison of YAlO_3_ with regular and distorted octahedra up to *p* = 8 GPa; (*c*) Extrapolation of YAlO_3_ with regular and distorted octahedra up to *p* = 52 GPa. Dashed lines indicate pressures for which structural data are quoted in Table 10 ▸.

200 equally spaced pressures were taken within the range up to 125 GPa and *a*, *b*, *c* values calculated from the above Birch–Murnaghan constants. Values of *x*(O1), *y*(O1), *x*(O2), *y*(O2) and *z*(O2) were allowed to vary within the core *Excel* Solver functionality for space group *Pbnm* (Fig. 7 ▸), so as to generate regular octahedra. The resulting variation of parameters η_*A*_ and η_*B*_ with pressure is shown in Fig. 11 ▸(*a*).

Parameter η_*A*_ decreases from 0.9576 at 0 GPa to 0.9529 at 125 GPa, which indicates an increase in 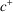 tilting. By comparison, η_*B*_ increases from 1.0649 at 0 GPa to 1.0939 at 125 GPa, indicating an increase in 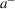 tilting. The effect of pressure is to drive the structure ever further into the *Pbnm* phase field. It is likely that the imminent phase transition to post-perovskite at 125 GPa is triggered by the strong O⋯O repulsions that will be associated with this degree of octahedral tilting.

### 4.2. Simulation of YAlO_3_ at pressures up to 52 GPa

Ross *et al.* (2004 ▸) elucidated the response of the perovskite compound YAlO_3_ at pressures up to 8 GPa by providing eight structural refinements of a synthetic single crystal. A *decrease* in octahedral tilting with pressure was found, which was ascribed to the AlO_6_ octahedral compressibility being greater than that of the YO_12_ site. It follows that an approach towards cubic symmetry will occur, similar to the *T*-series with *A* ion perturbation in §3.1. The Birch–Murnaghan constants to third order quoted by Ross *et al.* (2004 ▸) were used to generate unit-cell parameters at different pressures, as in §4.1. Two alternative simulations were carried out for pressures up to 8 GPa, the first assuming regular octahedra as in §4.1, and the second exploiting the octahedral distortions reported by Ross *et al.* (2004 ▸) from an analysis of their structural refinements. For this purpose, the linear, decreasing trends in the three different Al—O bond lengths with increasing pressure observed by them were translated into linear relationships for PCRO parameters *a*_1_ to *a*_3_. In addition, linear relationships for PCRO angle parameters θ_23_, θ_31_ and θ_12_ were derived [equations (8)].

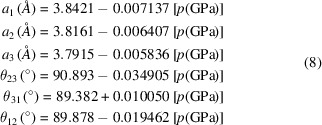

Solver-based refinements in *Pbnm* were carried out at equally spaced pressure values, the spacing determined by covering the pressure range from 0 to 60 GPa with 200 values. Parameters *x*(O1), *y*(O1), *x*(O2), *y*(O2) and *z*(O2) were allowed to vary in refinements with end-point determined by the minimum deviation from conditions (8). The deviation was observed to range between 0.001 and 0.050%.

The results of both simulations are shown in Fig. 11 ▸(*b*) and in that part of Fig. 11 ▸(*c*) with the blue-shaded background. The slope of both η_*B*_ curves is negative, compared to the positive slope of η_*B*_ for MgSiO_3_. The slope of η_*A*_ for the simulation with regular octahedra is positive, compared to the negative slope for MgSiO_3_. The opposite behaviour to MgSiO_3_ is shown by YAlO_3_, which shows a gradual progression away from *Pbnm* towards higher symmetry. The curve for η_*A*_ for distorted octahedra has a shallow minimum at ∼3.4 GPa, which is more clearly seen in Fig. 11 ▸(*c*). Thereafter η_*A*_ rises, as for undistorted octahedra.

In a computational experiment, the pressure was allowed to rise towards 60 GPa, with unit-cell constants calculated from the Birch–Murnaghan constants of Ross *et al.* (2004 ▸) for the pressure range up to 8 GPa. Fig. 11 ▸(*c*) shows that the curvature of both pairs of curves changes sign as the octahedra change from regular to distorted. Since the constraints in equations (8) only apply up to 8 GPa, it became increasingly difficult to reach low deviations at the end-points of the refinements. The maximum deviation amounted to 1.08%, which was obtained at the highest pressure.

The ascending curve for η_A_ with distorted octahedra in Fig. 11 ▸(*c*) reaches zero at a pressure of ∼41.5 GPa. This would be consistent with a phase transition to *Ibmm* [*cf.* Figs. 10 ▸(*a*) and 10 ▸(*b*)]. By comparison, the η_*B*_ curve is the first to reach zero in the simulation with regular octahedra. The corresponding pressure is ∼51.6 GPa. This would be consistent with a phase transition to *I*4/*mcm* or *P*4/*mbm* [*cf.* Fig. 10 ▸(*d*)]. The asymptotic approach of the η_*B*_ curve to 1 for regular octahedra gives rise to a broad pressure range in which *Cmcm* could be stabilized as well as *Pbnm* prior to this phase transition. This phenomenon has been observed in NaTaO_3_ by Knight & Kennedy (2015 ▸) over a broad *temperature* range, as discussed in §3.1.4. Assuming pattern similarity, a phase change to *P*4/*mbm* would be anticipated at 51.6 GPa. More generally, the simulations of YAlO_3_, both with and without octahedral distortion, indicate how this distortion is expected to have a direct effect on the sequence of phase transitions and the pressures at which they occur.

In summary, the analysis of (*p–T–X*)-induced phase transitions in terms of η_*A*_ and η_*B*_ parameters is a stimulus to further, targeted experimental work on the systems that have been analysed and simulated in §3.1, §4.1 and §4.2.

### 4.3. Crystal structures generated in the high-pressure simulations

A by-product of the simulations in §3.1 is the generation of full sets of oxygen ion coordinates. Table 10 ▸ contains these data corresponding to the conditions denoted by dashed lines in Figs. 11 ▸(*a*) and 11 ▸(*c*). The space group is *Pbmn* with the B ions in 4*b* positions. The full set of associated structural parameters is also quoted below the line in the table.

In all cases, the effect of increasing pressure is to reduce *V_A_* and *V_B_*. However, the ratio *V_A_*/*V_B_* is reduced in MgSiO_3_ and increased in YAlO_3_. This signifies movement further into the *Pbnm* phase field in the former case [Fig. 11 ▸(*a*)] and movement away towards higher symmetry in the latter [Fig. 11 ▸(*c*)]. This is borne out by the changes in inclination angles θ*_x_*, θ*_x_*, θ*_x_*, tilt angles ϕ_a_, ϕ_c_ and tilt-related parameters η_*A*_, η_*B*_.

### 4.4. Analysis of (La_1–*x*_Nd*_x_*)GaO_3_ structures at pressures of up to 12 GPa

Apart from the above simulations, the structural parameters used in this work are applied to the structural refinements of Angel *et al.* (2007 ▸) for this 3:3 perovskite solid solution. In carrying out *A*-ion substitutional perturbations, it was found that a phase transition from *Pbnm* to 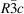 takes place under pressure for *x* values up to 0.20, but not for *x* = 0.62 or *x* = 1. The question arises as to whether a particular pattern of structural evolution within the *Pbnm* phase is associated with a phase transition to 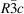 . Data for the *Pbmn* and 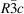 phases at their lowest and highest investigated pressures for a given *x* value are given in Table 11 ▸.

The direct influence of the *A*-ion perturbations is seen in the values of *V_A_*, which decrease downwards in the table with increasing *x* for the *p* = 0.0001 GPa values. Since *V_B_* remains approximately constant, a parallel trend of decreasing *V_A_*/*V_B_* ratio with increasing *x* is generally observed. This is consistent with greater stabilization within the *Pbnm* phase field. Pressure induces the opposite trend to raising *x*, since the *V_A_*/*V_B_* ratios for maximum pressures within the *Pbnm* phase field are uniformly higher than at atmospheric pressure. A parallel increase in η_*A*_ and decrease in η_*B*_ is observed. At lower *x* values up to 0.20, which are associated with reduced *Pbnm* stabilization, the increased pressure induces a phase transition to 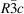 . Angel *et al.* (2007 ▸) report the following approximate pressures for this phase transition: *x* = 0: 2.2 GPa; *x* = 0.06: 5.5 GPa; *x* = 0.12: 7.8 GPa; *x* = 0.20: 12 GPa. The expected cross-correlations between parameter pairs η_*A*_ ↔ ϕ_c_ and η_*B*_ ↔ ϕ_a_ are observed within the *Pbnm* phase field: the greater the magnitude of the deviations of η_*A*_ and η_*B*_ from one, the larger the 〈ϕ_c_〉 and 〈ϕ_a_〉 angles. It is proposed that the observed fall in 〈ϕ_c_〉 to ∼4.2–4.4° with increasing pressure is the principal driving force for the phase transitions to 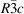 . After the transitions for *x* = 0 and *x* = 0.12, *i.e.* within the 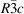 phase field, the ϕ_a_ tilt angle is larger and approximately equal to the mean tilt angle (〈ϕ_a_〉 + 〈ϕ_c_〉)/2 in the *Pbnm* field beforehand. For *x* = 0.62 and *x* = 1.00, the critical range of ϕ_c_ between 4.2 and 4.4° is not reached at the pressures investigated, so that these compounds remain stabilized in space group *Pbnm*.

### 4.5. Structural parameters for YAl_0.25_Cr_0.75_O_3_ with locked octahedral tilting

Ardit *et al.* (2017 ▸) subsequently took up the theme of locked octahedral tilting in orthorhombic 3:3 perovskites (*A* ion +3; *B* ion +3) by reference to the solid solution YAl_0.25_Cr_0.75_O_3_ in space group *Pbnm*. Although in general agreement with Zhao *et al.* (2004 ▸) and Angel *et al.* (2005 ▸) in assuming compressibility ratios β(*A*O_12_)/β(*B*O_6_) < 1 for a 3:3 perovskite and > 1 for a 2:4 perovskite, their compound showed a compressibility ratio approximately equal to one (Table 12 ▸).

In spite of consistently falling 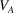 and 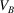 values with increasing pressure, the *V_A_*/*V_B_* ratio remains approximately constant, as do parameters 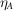 , 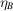 , 〈ϕ_c_〉 and 〈ϕ_a_〉. Discrepancies are observed between the values of the tilt angles calculated according to §2.3 and the values quoted by Ardit *et al.* (2017 ▸) without declaring the method of calculation. This is not unexpected, as discussed in §2.4. Since values of 〈ϕ_c_〉 and 〈ϕ_a_〉 calculated according to §2.3 show the expected cross-correlations between parameter pairs η_A_↔ϕ_c_ and η_B_↔ϕ_a_, indirect support is given for the correctness of these calculations.

## 5. Discussion

The ability of the method to analyse experimental structural data and the sequences of phase transitions between space groups has been demonstrated in §3 and §4. By comparison with the group-theoretical approach, it has not been necessary to relate these explicitly to the cubic aristotype, since deviations of *V_A_*/*V_B_* from the limiting value of 5 in the aristotype provide a direct indication of how far away a given structure is from the aristotype. Parameters *V_A_*, *V_B_* and *V_A_*/*V_B_* continue to yield valuable insight, for example in Table 11 ▸. However, the direct calculation of Glazer tilt angles ϕ_c_ and [ϕ_a_, ϕ_b_] and associated derived parameters η_*A*_ and η_*B*_ has allowed a deeper analysis of the structural factors leading to phase transitions than a consideration of these volumes alone. The significance of the generalized algorithms introduced here for the Glazer tilt angles may be assessed by reference to Wang & Angel (2011 ▸): ‘…the decomposition of a perovskite structure including tilted and distorted octahedra by geometric analysis does not result in an unambiguous definition of the Glazer (1972 ▸) tilts and the problem is more acute in perovskites with lower space-group symmetries’. These authors noted further that ‘unambiguous expressions for both the Glazer tilts and their relationship to the *V_A_*/*V_B_* ratio are still to be determined explicitly for each space group, and in a general form’. These observations led Wang & Angel (2011 ▸) to resort to group-theoretical methods in order to relate the amplitudes of symmetry-adapted modes to *V_A_*/*V_B_* ratio. The current work, by comparison, has remained strictly based on unit-cell parameters and atomic coordinates. Although it has not provided generalized analytical expressions for these tilt angles, it has led to two generalized algorithms (*a*) for all centrosymmetric space groups apart from 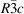 and (*b*) for space group 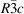 (and more generally, triclinic space groups). An analytical link between ϕ_c_, [ϕ_a_, ϕ_b_] and *V_A_*/*V_B_* has not been provided, since inclination angles 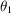 to 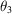 are more suitable for this purpose [equation (1)]. However, the linked-cell implementation within *Excel* allows the empirical derivation of numerical relationships between Glazer tilt angles and *V_A_*/*V_B_*.

The methodological innovations of the current work and their benefits are summarized in Table 13 ▸.

The question of the relative stability of alternative perovskite phases has been addressed by several workers employing quantum-mechanical methods (Zagorac *et al.*, 2014 ▸) as well as the semi-empirical bond-valence method. Woodward (1997*b* ▸) initiated this discussion by evaluating the ionic and covalent bonding in perovskites and analysing the conditions of stabilization of favoured tilt systems 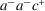 , 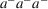 and 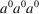 . In later work (Lufaso & Woodward, 2001 ▸), an algorithm was developed to minimize the so-called global instability index (GII) in alternative tilt systems, this being the r.m.s. deviation between calculated bond valences and ideal cationic valences. The bond-valence method was also the favoured approach of Zhao *et al.* (2004 ▸) in rationalizing the relative compressibilities of the *A*O_12_ and *B*O_6_ polyhedra in perovskites. It led to a clear differentiation in behaviour between 2:4 and 3:3 perovskites, which has remained a feature of experimentally led investigations of perovskites under pressure, for example by Ardit *et al.* (2017 ▸).

The potential of direct transformation of crystallographic data into structural parameters for the analysis of sequences of phase transitions has been demonstrated in this work. The set of structural parameters has been extended and a baseline provided for future investigations of non-centrosymmetric perovskites. The intention is to promote more detailed interaction between experimental crystallography and modelling in developing new materials by atomic and molecular design.

## 6. Related literature

The following references are cited in the supporting information: Hahn (1995 ▸), Williams (1971 ▸).

## Supplementary Material

Sections S1-S3.2 and Tables S1-S19. DOI: 10.1107/S2052520621012713/ra5104sup1.pdf

This Excel file is referred to in the main text and in the supporting information pdf file. DOI: 10.1107/S2052520621012713/ra5104sup2.xlsx

## Figures and Tables

**Table 1:** Calculation of analytical fractional coordinates of the six oxygen anions coordinating the titanium ion in CaTiO_3_ located at 0,½,0

Atom	Numerical fractional coordinates			Cartesian coordinates			Analytical fractional coordinates		
	*x*	*y*	*z*	*x*_C_	*y*_C_	*z*_C_	*x*	*y*	*z*
Ti (4*b*)	0	0.5	0	0.0000	2.7140	0.0000	0	½	0
O a (8*f*)	−0.2113	0.7891	0.0375	−1.1349	4.2832	0.2860	−*x*(O2) + ½	*y*(O2) + ½	*z*(O2)
O b (8*f*)	0.2113	0.2109	−0.0375	1.1349	1.1448	−0.2860	*x*(O2) − ½	−*y*(O2) + ½	−*z*(O2)
O c (8*f*)	−0.2887	0.2891	0.0375	−1.5506	1.5692	0.2860	*x*(O2) − 1	*y*(O2)	*z*(O2)
O d (8*f*)	0.2887	0.7109	−0.0375	1.5506	3.8588	−0.2860	−*x*(O2) + 1	−*y*(O2) + 1	−*z*(O2)
O e (4*c*)	−0.0708	0.5170	−0.25	−0.3803	2.8063	−1.9067	−*x*(O1)	−*y*(O1) + 1	−¼
O f (4*c*)	0.0708	0.4830	0.25	0.3803	2.6217	1.9067	*x*(O1)	*y*(O1)	¼

**Table 2:** The three vectors defining the PCRO with mid-point 0,½,0 in space group *Pbnm*

		Cartesian components			Nearest pseudocubic axes	
Stalk	PCRO vector	*X*	*Y*	*Z*	Pseudocubic	Orthorhombic
O b ← O a	**a**_1_	*a*(2*x*(O2) − 1)	− 2*by*(O2)	− 2*cz*(O2)	*x*_PC_	]0{\bar 1}0]
O d ← O c	**a**_2_	−2*a*(*x*(O2) − 1)	−*b*(2*y*(O2) − 1)	−2*cz*(O2)	*y*_PC_	[110]
O f ← O e	**a**_3_	2*ax*(O1)	*b*(2*y*(O1) − 1)	*c*/2	*z*_PC_	[001]

**Table 3:** Summary of Solver refinements by space group corresponding to structures with regular octahedra The octahedral and unit-cell volumes are identical to those in the reference structures.

Space group	*Pbnm* (B: 4*b*)	*Pbnm* (B: 4*a*)		*Cmcm*		*Ibmm*		*P*4/*mbm*	*P*4_2_/*nmc*	*I*4/*mcm*		R{\bar 3}c
Reference system	CaTiO_3_	LaCr_0.7_Ni_0.3_O_3_		NaNbO_3_ at 848 K		BaPbO_3_ at 300 K		NaNbO_3_ at 888 K	CaMnO_3_	CaTiO_3_ at 1523 K		La(Cr_0.2_Ni_0.8_)O_3_
Reference ICSD	162908	173471		192404		154038		280100	670342	162919		173475
*a*_1_(refined) (Å)	3.9014	3.9535		3.9495		4.3480		3.9500	3.8074	3.9025		3.8952
*a* (Å)	5.33816	5.46101		7.85139		6.08315		5.56480	7.52285	5.47004		5.42241
*b* (Å)	5.44997	5.57887		7.85672		6.14899						
*c* (Å)	7.64264	7.73997		7.89355		8.60287		3.94998	7.43089	7.80495		13.49346
*x*(O1)	0.07274	0.57385		0.27596		0.05216		0	0.03909	0		0.55170
*y*(O1)	0.48876	0.49521		0		0		0	−*x*(O1)	0		0
*z*(O1)	¼	¼		0		¼		½	¼	¼		¼
*x*(O2)	0.70974	0.23308		0		¼		0.22810	¼	0.21650		
*y*(O2)	0.28863	0.26621		0.22407		¼		0.72810	0.00306	0.71650		
*z*(O2)	0.03637	0.03692		0.00921		−0.02608		0	0.96043	0		
*x*(O3)				0.25921					¼			
*y*(O3)				0.25096					*y*(O2)			
*z*(O3)				¼					−*z*(O2) + {1 \over 2}			
Method	§2.3	§2.3	a)	§2.3	b)	§2.3	d)		§2.3	§2.3	c)	§2.3
ϕ_a_ (°)	8.52	8.47	11.91			6.37	8.72		7.13			5.90
ϕ_a,refined_ (°)	8.29	8.33	11.80			5.92	8.39		8.89			5.90
ϕ_b_ (°)				2.11	4.15				6.02			
ϕ_b,refined_ (°)				2.12	2.11				8.89			
ϕ_c_ (°)	8.84	4.90	4.90	5.38	5.38			5.01	9.34	7.63	7.63	
ϕ_c,refined_ (°)	8.96	3.79	3.79	5.92	5.92			5.01	0.70	7.63	7.63	
θ_3_ (°)		10.97				7.87						
θ_3,refined_ (°)		11.80				8.39						

**Table 4:** Summary of inclination angles, PCRO parameters and degrees of freedom by space group

Space group	*Pbnm*	*Cmcm*	*Ibmm*	*I*4/*mcm* and *P*4/*mbm*	*P*4_2_/*nmc*	R{\bar 3}c	Pm{\bar 3}m
3-axis in equation (1)	*z*	–	*z*	*z*	*z*	–	–
Inclination angles	{{{\theta}}_1} \ne 0	{{{\theta}}_1} \ne 0	{{{\theta}}_1} = {{{\theta}}_2} \ne 0	{{{\theta}}_1} \ne 0	{{{\theta}}_1} \ne 0	{{{\theta}}_1} = {{{\theta}}_2} = {{{\theta}}_3} \ne 0	{{{\theta}}_1} = {{{\theta}}_2} = {{{\theta}}_3} = 0
	{{{\theta}}_2} \ne 0	{{{\theta}}_2} \ne 0		{{{\theta}}_2} \ne 0	{{{\theta}}_2} \ne 0		
	{{{\theta}}_3} \ne 0	{{{\theta}}_3} \ne 0	{{{\theta}}_3} \ne 0	{{{\theta}}_3} = 0	{{{\theta}}_3} \ne 0		
PCRO parameter equalities			{a_1} = {a_3}	{a_1} = {a_2}		{a_1} = {a_2} = {a_3}	{a_1} = {a_2} = {a_3}
			{{{\theta}}_{23}} = {{{\theta}}_{12}}	{{{\theta}}_{23}} = {{{\theta}}_{31}} = {{{\theta}}_{12}} = 90^\circ		{{{\theta}}_{23}} = {{{\theta}}_{31}} = {{{\theta}}_{12}}	{{{\theta}}_{23}} = {{{\theta}}_{31}} = {{{\theta}}_{12}} = 90^\circ
Tilt system	{a^ - }{a^ - }{c^ + }	{a^0}{b^ - }{c^ + }	{a^ - }{a^ - }{c^0}	{a^0}{a^0}{c^ - } and {a^0}{a^0}{c^ + }	{a^ + }{b^ + }{c^ - } or {a^ + }{a^ + }{c^ - }	{a^ - }{a^ - }{a^ - }	*a^0^a^0^a^0^*
							
*N*(UC)	3	3	3	2	2	2	1
*N*(*X*)	5	5	2	1	5	1	0
*N*(tilt)	2	2	1	1	2	1	0
*N*(PCRO)	6	6	4	2	5	2	1
							
*N*(*A*)	2	2	1	0	0	0	0
*N*(*B*)	0	0	0	0	0	0	0

**Table 5:** Analytical expressions for structural parameters η_*A*_ and η_*B*_ to quantify {c^ \pm } and {a^ \pm }{b^ \pm } tilting

Space group	*N*(tilt)	η_*A*_	η_*B*_
*Pbnm* (*B* in 4*b*)	2	(1 − [3 − 4*x*(O2)][4*y*(O2) − 1])	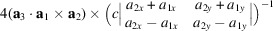
*Pbnm* (*B* in 4*a*)	2	([1 + [4*x*(O2) − 1][4*y*(O2) − 1])	As for *Pbnm* (*B* in 4*b*)
*Cmcm*	2	16*x*(O1)*y*(O2)	As for *Pbnm* (*B* in 4*b*)

*Ibmm*	1	1	As for *Pbnm* (*B* in 4*b*)
*P*4/*mbm*, *I*4/*mcm*	1	8*x*(O2)(1− 2*x*(O2))	1
*P*4_2_/*nmc*	3	2*a*,2*b*: 4(−{3\over 2} + 2*y*(O3))({1 \over 2}+ 2*y*(O2))	As for *Pbnm* (*B* in 4*b*)
		4*d*: 4({{5}\over{2}} − 2*y*(O3))({{1}\over{2}} − 2*y*(O2))	
R{\bar 3}c	1	4*x*(O)(1 − *x*(O))	–

**Table 6:** Structural parameters for two solid solutions spanning space group *Ibmm* at room temperature

Composition	ICSD	Space group	η_*A*_	η_*B*_	*V_A_* (Å^3^)	*V_B_* (Å^3^)	*V_A_*/*V_B_*	λ	σ
SrSnO_3_	190602	*Pbnm*	0.9847 (1)	1.0313 (3)	54.181 (5)	11.454 (4)	4.730 (2)	0.0023 (4)	0.0104 (8)
Sr_0.8_Ba_0.2_SnO_3_	190611	*Pbnm*	0.9935 (2)	1.0236 (3)	55.10 (6)	11.42 (1)	4.824 (2)	0.0052 (7)	0.0195 (9)
Sr_0.6_Ba_0.4_SnO_3_	190610	*Ibmm*	1	1.0147 (2)	56.02 (7)	11.40 (2)	4.913 (1)	0.0004 (3)	0.0091 (8)
Sr_0.4_Ba_0.6_SnO_3_	190609	*I*4/*mcm*	0.9960 (1)	1	56.9 (1)	11.43 (2)	4.9758 (6)	0.0000 (4)	0
Sr_0.2_Ba_0.8_SnO_3_	190608	Pm{\bar 3}m	1	1	57.52 (8)	11.50 (2)	5	0	0
BaSnO_3_	190601	Pm{\bar 3}m	1	1	58.071 (4)	11.6142 (8)	5	0	0
SrHfO_3_	89383	*Pbnm*	0.9856 (6)	1.034 (1)	55.64 (2)	11.79 (2)	4.719 (8)	0.003 (2)	0.013 (1)
Sr_0.8_Ba_0.2_HfO_3_	55746	*Pbnm*	0.996 (2)	1.029 (4)	57.16 (6)	11.89 (6)	4.81 (3)	0.033 (6)	0.058 (9)
Sr_0.6_Ba_0.4_HfO_3_	55747	*Ibmm*	1	1.026 (1)	57.97 (1)	11.96 (1)	4.848 (7)	0.0058 (5)	0.039 (3)
Sr_0.4_Ba_0.6_HfO_3_	55748	*I*4/*mcm*	0.98755	1	58.88 (1)	11.95 (1)	4.926 (5)	0.0013 (2)	0
Sr_0.2_Ba_0.8_HfO_3_	55749	Pm{\bar 3}m	1	1	59.7948 (9)	11.9590 (2)	5	0	0
BaHfO_3_	–	Pm{\bar 3}m	1	1	60.2909 (4)	12.0582 (1)	5	0	0

**Table 7:** Focus on the *Cmcm* phases of NaTaO_3_ and NaNbO_3_ with comparative data for neighbouring *Pbnm* and *P*4/*mbm* phases

Compound	ICSD	Temperature (K)	*V_A_/V_B_*	η_*A*1_	η_*A*2_	η_*B*_	Reference
*Pbnm*							
NaTaO_3_	150430	293	4.74 (2)	0.988 (2)		1.032 (3)	Mitchell & Liferovich (2004 ▸)
NaNbO_3_	192400	293	4.802 (5)	0.9868 (6)		1.0206 (6)	Mitchell *et al.* (2014 ▸)
*Cmcm*							
NaTaO_3_	280099	803	4.899 (2)	0.987 (3)	0.994 (3)	1.0076 (2)	Darlington & Knight (1999 ▸)
NaTaO_3_	241445	778	4.891 (2)	1.002 (2)	0.977 (2)	1.0076 (2)	Knight & Kennedy (2015 ▸)
NaTaO_3_ †	239691	783	4.889 (3)	0.991 (5)	0.989 (5)	1.0087 (3)	Arulnesan *et al.* (2016 ▸)
NaNbO_3_	192404	848	4.928 (3)	1.051 (3)	0.933 (3)	1.0041 (4)	Mitchell *et al.* (2014 ▸)
NaNbO_3_	192405	873	4.940 (2)	1.063 (3)	0.924 (3)	1.0033 (3)	Mitchell *et al.* (2014 ▸)
*P*4/*mbm*							
NaTaO_3_	88377	843	4.945 (1)	0.9907 (2)			Kennedy, Prodjosantoso *et al.* (1999 ▸)
NaNbO_3_	192406	898	4.9687 (7)	0.9948 (1)		1	Mitchell *et al.* (2014 ▸)

† Doped with 1 mol% K.

**Table 8:** Structural data relevant to the *Pbnm* → R{\bar 3}c transition at *x* ∼ 0.7 in LaCr_1–*x*_Ni*_x_* O_3_

Composition	ICSD	Space group	η_*A*_	η_*B*_	〈ϕ_c_〉 (°)	〈ϕ_a_〉 (°)	(〈ϕ_a_〉 + 〈ϕ_c_〉)/2 (°)	*V*_*A*_ (Å^3^)	*V*_*B*_ (Å^3^)	*V*_*A*_/*V*_*B*_
*x* = 0	173469	*Pbnm*	0.9925 (1)	1.0355 (7)	5.02 (3)	8.5 (2)	6.78 (8)	48.38 (1)	10.182 (7)	4.751 (4)
*x* = 0.3	173471	*Pbnm*	0.9836 (4)	1.0278 (9)	8.67 (5)	5.4 (2)	7.05 (8)	48.28 (1)	10.178 (9)	4.744 (5)
*x* = 0.6	173472	*Pbnm*	0.9900 (1)	1.0301 (8)	6.13 (4)	6.5 (2)	6.32 (10)	48.388 (9)	10.152 (8)	4.766 (5)
*x* = 0.7	173473	*Pbnm*	0.9983 (3)	1.0254 (5)	6.36 (3)	4.64 (9)	5.50 (5)	48.204 (9)	9.956 (6)	4.841 (3)
*x* = 0.7	173474	R{\bar 3}c	0.9891 (1)			5.97 (3)		47.453 (5)	9.867 (4)	4.809 (2)
*x *= 0.8	173475	R{\bar 3}c	0.9893 (2)			5.90 (5)		47.414 (7)	9.850 (5)	4.814 (3)
*x* = 0.9	173476	R{\bar 3}c	0.9897 (1)			5.79 (3)		47.29 (1)	9.810 (4)	4.820 (2)
*x *= 1	173477	R{\bar 3}c	0.9900 (1)			5.72 (3)		46.778 (3)	9.696 (2)	4.825 (1)

**Table 9:** Validation of Birch–Murnaghan (B-M) constants for use at pressures of up to to ∼125 GPa†

Method/calculation type	*p* (GPa)	*a* (Å)	*b* (Å)	*c* (Å)	*V*_uc_ (Å^3^)
Murakami XRD	109	4.325	4.579	6.308	124.9
Murakami MD	109	4.403	4.574	6.410	129.1
Extrapolated B-M	109	4.365	4.559	6.315	125.7
Fiquet synchrotron XRD	40.66	–	–	–	143.26
Extrapolated B-M	40.71	4.571	4.743	6.606	143.22

† *V*_uc_: unit-cell volume.

**Table 10:** Crystallographic and structural parameters generated in the simulations of MgSiO_3_ and YAlO_3_

Compound	MgSiO_3_	MgSiO_3_	YAlO_3_	YAlO_3_	YAlO_3_	YAlO_3_
Pressure (GPa)	109	125	8	8	31.5	31.5
Octahedra	Regular	Regular	Regular	Distorted	Regular	Distorted
*a* (Å)	4.3649	4.3278	5.1285	5.1285	5.0308	5.0308
*b* (Å)	4.5592	4.5263	5.2443	5.2443	5.0751	5.0751
*c* (Å)	6.3151	6.2628	7.2899	7.2899	7.1349	7.1349
*x*(O1)	0.1067	0.1083	0.0756	0.0810	0.0470	0.0604
*y*(O1)	0.4780	0.4775	0.4925	0.4810	0.4965	0.4934
*x*(O2)	0.6936	0.6933	0.7241	0.7054	0.7309	0.7241
*y*(O2)	0.3017	0.3019	0.2748	0.2940	0.2687	0.2758
*z*(O2)	0.0533	0.0542	0.0378	0.0420	0.0235	0.0295
γ_PC_ (°)	92.49	92.57	91.28	91.28	90.50	90.50
*V_A_* (Å^3^)	25.439	24.816	40.386	40.127	37.772	37.651
*V_B_* (Å^3^)	5.979	5.855	8.630	8.889	7.769	7.890
*V_A_*/*V_B_*	4.255	4.239	4.680	4.514	4.862	4.772
θ*_x_* (°)	16.89	17.05	10.27	13.64	6.88	8.90
θ*_y_* (°)	16.89	17.05	10.27	13.71	6.88	8.91
θ*_z_* (°)	16.79	17.03	12.06	13.19	7.58	9.72
〈ϕ_c_〉 (°)	12.19	12.24	5.79	10.05	4.32	5.90
〈ϕ_a_〉 (°)	12.05	12.25	8.55	9.48	5.36	6.72
η_*A*_	0.9534	0.9529	0.9898	0.9686	0.9943	0.9893
η_*B*_	1.0910	1.0939	1.0457	1.0549	1.0177	1.0285
*a*_1_ (Å)	3.2981	3.2750	3.7272	3.7845	3.5989	3.6193
*a*_2_ (Å)	3.2981	3.2750	3.7272	3.7647	3.5989	3.6138
*a*_3_ (Å)	3.2981	3.2750	3.7272	3.7437	3.5989	3.6194
θ_23_ (°)	90	90	90	90.61	90	89.85
θ_31_ (°)	90	90	90	89.46	90	89.75
θ_12_ (°)	90	90	90	89.69	90	89.71
λ_PC_	0	0	0	0.0037	0	0.0007
σ_PC_	0	0	0	0.012	0	0.004

**Table 11:** Structural parameters for (La_1–*x*_Nd*_x_*)GaO_3_ in space groups *Pbnm* and R\bar 3c

*x*	Space group	*p* (GPa)	ICSD	η_*A*_	η_*B*_	〈ϕ_c_〉 (°)	〈ϕ_a_〉 (°)	(〈ϕ_a_〉 + 〈ϕ_c_〉)/2 (°)	*V*_*A*_ (Å^3^)	*V*_*A*_ (Å^3^)	*V*_*A*_/*V*_*B*_
0	*Pbnm*	0.0001	160233	0.9926 (2)	1.0406 (7)	4.90 (8)	8.5 (1)	6.69 (7)	48.653 (8)	10.299 (8)	4.724 (4)
	*Pbnm*	2.038 (10)	160235	0.9939 (2)	1.0382 (7)	4.46 (7)	8.3 (1)	6.39 (7)	48.136 (7)	10.146 (7)	4.744 (4)
	R\bar 3c	2.356 (7)	160265	0.9868 (3)			6.56 (7)		48.024 (9)	10.066 (8)	4.771 (5)
	R\bar 3c	8.127 (23)	160270	0.9872 (3)			6.45 (7)		46.745 (8)	9.782 (8)	4.779 (4)
0.06	*Pbnm*	0.0001	160236	0.9902 (4)	1.044 (2)	5.7 (1)	9.1 (3)	7.4 (1)	48.50 (2)	10.33 (2)	4.693 (9)
	*Pbnm*	5.30 (5)	160240	0.9947 (4)	1.042 (2)	4.2 (2)	9.1 (3)	6.6 (2)	47.18 (2)	9.98 (2)	4.73 (1)
0.12	*Pbnm*	0.0001	160241	0.9907 (4)	1.040 (1)	5.5 (1)	8.5 (2)	7.0 (1)	48.55 (1)	10.29 (1)	4.716 (7)
	*Pbnm*	6.437 (7)	160243	0.9939 (3)	1.039 (1)	4.4 (1)	8.5 (2)	6.5 (1)	46.96 (1)	9.91 (1)	4.741 (6)
	R{\bar 3}c	8.020 (2)	160271	0.9875 (5)			6.4 (1)		46.65 (2)	9.75 (1)	4.783 (9)
	R{\bar 3}c	9.496 (13)	160272	0.9872 (4)			6.46 (9)		46.37 (1)	9.71 (1)	4.778 (6)
0.20	*Pbnm*	0.0001	160244	0.9884 (4)	1.0425 (7)	6.2 (1)	8.7 (1)	7.45 (7)	48.404 (9)	10.323 (8)	4.689 (5)
	*Pbnm*	8.671 (7)	160248	0.9931 (3)	1.042 (1)	4.7 (1)	8.8 (2)	6.79 (9)	46.38 (1)	9.83 (1)	4.720 (6)
0.62	*Pbnm*	0.0001	160250	0.9807 (5)	1.049 (1)	7.9 (1)	9.1 (1)	8.52 (8)	47.72 (1)	10.35 (1)	4.611 (6)
	*Pbnm*	9.432	160256	0.9842 (7)	1.046 (1)	7.2 (2)	8.9 (2)	8.0 (1)	45.60 (1)	9.81 (1)	4.648 (7)
1.00	*Pbnm*	0.0001	160257	0.9749 (5)	1.0537 (9)	9.00 (9)	9.6 (1)	9.28 (8)	47.18 (1)	10.36 (1)	4.555 (6)
	*Pbnm*	8.292 (9)	160264	0.9762 (4)	1.0514 (8)	8.78 (7)	9.4 (1)	9.11 (7)	45.324 (9)	9.909 (9)	4.574 (5)

**Table 12:** Structural parameters for eleven structural refinements of Ardit *et al.* (2017 ▸) on YAl_0.25_Cr_0.75_O_3_

*p* (GPa)	η_*A*_	η_*B*_	〈ϕ_c_〉 (°) (§2.3)	〈ϕ_a_〉 (°) (§2.3)	〈ϕ_c_〉 (°) (Ardit)	〈ϕ_a_〉 (°) (Ardit)	*V*_*A*_ (Å^3^)	*V*_*B*_ (Å^3^)	*V*_*A*_/*V*_*B*_
0.05 (5)	0.9557 (9)	1.082 (1)	11.9 (1)	11.7 (2)	12.0 (5)	16.6 (5)	43.52 (2)	10.10 (2)	4.309 (8)
1.44 (9)	0.9604 (4)	1.0868 (7)	11.24 (6)	12.32 (9)	12.7 (5)	16.6 (5)	43.238 (9)	10.030 (8)	4.311 (4)
3.32 (9)	0.9609 (5)	1.0862 (7)	11.18 (7)	12.32 (9)	12.7 (5)	16.5 (5)	42.892 (9)	9.938 (8)	4.316 (4)
4.93 (7)	0.9603 (5)	1.0871 (7)	11.25 (7)	12.36 (9)	12.8 (5)	16.6 (5)	42.561 (9)	9.878 (8)	4.309 (4)
6.36 (8)	0.9630 (5)	1.0890 (7)	10.88 (7)	12.67 (9)	13.1 (5)	16.5 (5)	42.321 (9)	9.812 (8)	4.313 (4)
8.48 (9)	0.9630 (5)	1.0890 (8)	10.88 (7)	12.67 (9)	13.1 (5)	16.6 (5)	41.954 (9)	9.727 (8)	4.313 (5)
10.54 (9)	0.9635 (6)	1.090 (1)	10.81 (9)	12.8 (1)	13.2 (5)	16.6 (5)	41.60 (1)	9.65 (1)	4.312 (6)
12.34 (7)	0.9580 (6)	1.086 (1)	11.57 (8)	12.2 (1)	12.6 (5)	16.5 (5)	41.28 (1)	9.59 (1)	4.303 (6)
14.40 (9)	0.9569 (8)	1.085 (1)	11.7 (1)	12.1 (2)	12.4 (5)	16.6 (5)	40.97 (1)	9.52 (1)	4.302 (7)
16.45 (9)	0.956 (1)	1.084 (2)	11.9 (2)	12.0 (2)	12.4 (5)	16.5 (5)	40.65 (2)	9.45 (2)	4.30 (1)
18.86 (9)	0.959 (2)	1.087 (3)	11.4 (3)	12.6 (3)	13.0 (5)	16.5 (5)	40.30 (3)	9.36 (3)	4.30 (2)

**Table 13:** Summary of innovations in the current work

Innovation	Benefits
Quantification of distortion of centro­symmetric octahedra via PCRO.	(*a*) Direct, concise visualization of octahedral distortion.
	(*b*) Variation of PCRO parameters rooted in space group symmetry.
	(*c*) New possibilities for simulating perovskite structures at extrapolated (*p–T–X*) conditions.
	(*d*) Many more visualizable structural parameters *cf.* Thomas (1998 ▸).
	(*e*) Upgradable to non-centrosymmetric octahedra, and so applicable to all perovskites and more widely to all crystal structures containing octahedra.
	(*f*) Provides summary distortion parameters λ and σ.
	
Generalized algorithms for calculating tilt angles [ϕ_a_, ϕ_b_] and ϕ_c_.	(*a*) Extension to space groups *Cmcm*, *P*4_2_/*nmc* and R{\bar 3}c not previously covered by analytical approximations.
	(*b*) Ability to calculate tilt angles in structures with distorted octahedra without approximation.
	
Structural parameters η_*A*_ and η_*B*_.	(*a*) η_*A*_ emphasizes the importance of *AX*_8_ inner polyhedra and focuses on octahedral tilting around the *z* (3) axis.
	(*b*) η_B_ focuses on tilting around the *x* (1) and *y* (2) axes.
	(*c*) Tracking structural evolution via η_*A*_ and η_*B*_ allows phase transitions between *Pbnm* and *Ibmm*, *Cmcm*, *I*4/*mcm* or *P*4/*mbm* to be rationalized and anticipated.
	(*d*) Improved integration with the tilt classification of Glazer (1972 ▸), and, by implication, group-theoretical methods.
	
Implementation in *Excel* Solver software environment.	(*a*) Reversibility of transformation between crystallographic and structural parameters.
	(*b*) The *Excel* file in the supporting information is a useful resource for calculating tilt angles and other parameters, as well as for refining crystallographic parameters under structural constraints.
	(*c*) Upgradable to other programming languages (Frontline Systems Inc., 2021 ▸).
	
Simulation of structural development at increasing pressure.	The assumption of regular or idealized distorted octahedra allows the prediction of crystal structure and associated parameters, *e.g.* η_*A*_, η_*B*_ from unit-cell parameters alone. The potential for modelling structures and phase transitions at high pressure is thereby increased.

## Footnotes

1 Since oxides are exclusively considered in this work, the notation ‘O1,O2…’ is used interchangeably with ‘*X*1,*X*2…’.

2 This activates a VBA macro, which calculates the values of structural parameters for the cells with a pink background. Although not included in the supporting information, the author is willing to supply an *xlsm* file containing VBA codes on request.

3 Polyhedral volume =  . *A*_i_ is area of face; *h*_i_ is perpendicular distance from face to common apex inside polyhedron.

4 Values of *K_T_* and *V*_0_ are in good agreement with the values quoted by Vanpeteghem *et al.* (2006 ▸): 253 (1) GPa and 162.51 (2) Å^3^.
