# Supplementary material for: Phase transitions and (p–T–X) behaviour of centrosymmetric perovskites: modelling with transformed crystallographic data
Source: Acta Crystallogr B Struct Sci Cryst Eng Mater. 2022 Jan 20;78(Pt 1):40–60. doi: 10.1107/S2052520621012713 (PMC8819707; doi:10.1107/S2052520621012713)
Supplement: Supplementary file 1 [file b-78-00040-sup1.pdf]

STRUCTURAL SCIENCE
CRYSTAL ENGINEERING
MATERIALS

Volume 78 (2022)

Supporting information for article:

Phase transitions and (*p*–*T*–*X*) behaviour of centrosymmetric perovskites: modelling with transformed crystallographic data

Noel W. Thomas

Supporting information for the article entitled

Phase transitions and (*p-T-X*) behaviour of centrosymmetric perovskites: modelling with transformed crystallographic data

Noel W. Thomas[a]*

[a]Werkstofftechnik Glas & Keramik, Hochschule Koblenz, Rheinstrasse 56, 56203 Hoehr-Grenzhausen, Germany

Correspondence email: thomas@hs-koblenz.de

S1. Special and general positions in space group *Pbnm* relevant for perovskites

The positions quoted in Table S1 have been derived from the positions quoted for *Pnma* in *International Tables for Crystallography* (Hahn 1995).

Table S1 Relevant positions in Space Group *Pbnm* for perovskites

Position	1	2	3	4	5	6	7	8
B (4*a*)	0,0,0	½,½,0	0,0,½	½,½,½				
B (4*b*)	½,0,0	0,½,0	½,0,½	0,½,½				
A & O1 (4*c*)	$x,y,¼$	$x+½,\bar{y}+½,¾$	$\bar{x},\bar{y},¾$	$\bar{x}+½,y+½,¼$				
O2 (8*d*)	x,y,z	$x+½,\bar{y}+½,\bar{z}$	$\bar{x},\bar{y},z+½$	$\bar{x}+½,y+½,\bar{z}+½$	$\bar{x},\bar{y},\bar{z}$	$\bar{x}+½,y+½,z$	$x,y,\bar{z}+½$	$x+½,\bar{y}+½,z+½$

S2. Derivation of analytical expressions for the three definitive PCRO vectors in different space groups

S2.1. Space group *Pbnm* with B-ions in 4*a* positions

The reference structure to be taken is of $LaCr_{0.7}Ni_{0.3}O_3$ (Yang (2008); ICSD 173471). The following unit cell parameters apply: a = 5.5094 Å; b = 5.4740 Å; c = 7.7536 Å. B-ions are located at 4*a* [0,0,0]. Oxygen O1 ions are located at 4*c* positions of the unit cell with x=0.5744 and y=0.4956. z(O1) has the fixed coordinate of ¼. O2 ions are located at 8*d* positions with x=0.2324, y=0.3082 and z=0.0236.

The following development follows the steps taken in §2.2.1 of the article with respect to the derivation of analytical coordinates. Tables S2, S4, S7, S9, S11, S13 and S15 are to be read from left to right, since

it is assumed that Cartesian coordinates are derived first, followed by numerical fractional coordinates and finally analytical fractional coordinates.

Table S2 Calculation of analytical fractional coordinates of the six octahedral anions coordinating the chromium ion located at 0,0,0

Atom	Cartesian coordinates			Numerical fractional coordinates			Analytical fractional coordinates		
	x_C	y_C	z_C	*x*	*y*	*z*	*x*	*y*	*z*
Cr (4*a*)	0.0000	0.0000	0.0000	0	0	0	0	0	0
O a *(8d)*	-1.4743	1.0499	-0.1830	-0.2676	0.1918	-0.0236	*x*(O2)-½	-*y*(O2)+½	-*z*(O2)
O b *(8d)*	1.4743	-1.0499	0.1830	0.2676	-0.1918	0.0236	-*x*(O2)+½	*y*(O2)-½	*z*(O2)
O c *(8d)*	-1.2804	-1.6871	-0.1830	-0.2324	-0.3082	-0.0236	-*x*(O2)	-*y*(O2)	-*z*(O2)
O d *(8d)*	1.2804	1.6871	0.1830	0.2324	0.3082	0.0236	*x*(O2)	*y*(O2)	*z*(O2)
O e *(4c)*	0.4099	0.0241	-1.9384	0.0744	0.0044	-0.25	*x*(O1)-½	-*y*(O1)+½	-¼
O f *(4c)*	-0.4099	-0.0241	1.9384	-0.0744	-0.0044	0.25	-*x*(O1)+½	*y*(O1)-½	¼

The three vectors for the PCRO are now formed in Table S3 by taking the differences in the analytical fractional coordinates given in Table S2 and forming Cartesian vectors by multiplying the vectors in fractional coordinates by the orthogonalization matrix $\begin{pmatrix} a & 0 & 0 \\ 0 & b & 0 \\ 0 & 0 & c \end{pmatrix}$. The three stalk vectors so formed are parallel to the edges of the PCRO.

Table S3 The three vectors defining the PCRO with mid-point 0,0,0 in space group *Pbnm*.

		Cartesian components			Nearest pseudocubic axes	
Stalk	PCRO vector	*X*	*Y*	*Z*	pseudocubic	orthorhombic
O b ← O a	$\boldsymbol{a}_1$	$-a(2x(\text{O2})-1)$	$b(2y(\text{O2})-1)$	$2cz(\text{O2})$	x_{PC}	$[1\bar{1}0]$
O d ← O c	$\boldsymbol{a}_2$	$2ax(\text{O2})$	$2by(\text{O2})$	$2cz(\text{O2})$	y_{PC}	[110]
O f ← O e	$\boldsymbol{a}_3$	$-a(2x(\text{O1})-1)$	$b(2y(\text{O1})-1)$	$c/2$	z_{PC}	[001]

The colour-coding of the cells signifies that vectors $\boldsymbol{a}_1$ and $\boldsymbol{a}_2$ interdependent. Both vectors are functions of the variables *a*,*b*,*c*, *x*(O2), *y*(O2) and *z*(O2).

S2.2. Space group *Cmcm*

The reference structure is of lueshite ($NaNbO_3$) at 848 K (Mitchell *et al.* (2014); ICSD 192404). The following unit cell parameters apply: *a* = 7.8679 Å; *b* = 7.8624 Å; *c* = 7.8713 Å. Atomic parameters for Nb and O are as follows: Nb: 8*d*; O1: 8*e*: *x*(O1) = 0.2809; O2: 8*f* : *y*(O2) = 0.2338, *z*(O2) = 0.0092; O3: 8*g*: *x*(O3) = 0.2771; *y*(O3) = 0.2579. Fixed oxygen coordinates are as follows: *y*(O1) = 0; *z*(O1) = 0; *x*(O2) = 0; *z*(O3) = ¼.

The octahedral cage coordinating the B-ion at ¼,¼,0 in the unit cell is taken, with the other seven octahedra in the cell generated by symmetry. Pseudocubic axes are set as follows: origin of coordinates at [¼, ¼,0]; x_{PC}: || [100]; y_{PC}: || [010]; z_{PC}: || [001]. Analytical fractional coordinates are calculated in Table S4.

Table S4 Calculation of analytical fractional coordinates of the six octahedral anions coordinating the niobium ion located at ¼,¼,0

Atom	Cartesian coordinates			Numerical fractional coordinates			Analytical fractional coordinates		
	X	*Y*	*Z*	*x*	*y*	*z*	*x*	*y*	*z*
Nb	1.9670	1.9656	0.0000	0.2500	0.2500	0.0000	¼	¼	0
O a (8*f*)	0.0000	1.8382	0.0724	0.0000	0.2338	0.0092	0	*y*(O2)	*z*(O2)
O b (8*f*)	3.9340	2.0930	-0.0724	0.5000	0.2662	-0.0092	½	-*y*(O2)+½	-*z*(O2)
O c (8*e*)	2.2101	0.0000	0.0000	0.2809	0.0000	0.0000	*x*(O1)	0	0
O d (8*e*)	1.7239	3.9312	0.0000	0.2191	0.5000	0.0000	-*x*(O1)+ ½	½	0
O e (8*g*)	1.7538	1.9035	-1.9678	0.2229	0.2421	-0.2500	-*x*(O3)+ ½	-*y*(O3)+ ½	-¼
O f (8*g*)	2.1802	2.0277	1.9678	0.2771	0.2579	0.2500	*x*(O3)	*y*(O3)	¼

The three vectors for the PCRO are now formed in Table S5 by taking the differences in the analytical fractional coordinates given in Table S4 and forming Cartesian vectors by multiplying the vectors in fractional coordinates by the orthogonalization matrix $\begin{pmatrix} a & 0 & 0 \\ 0 & b & 0 \\ 0 & 0 & c \end{pmatrix}$. The three stalk vectors so formed are parallel to the edges of the PCRO.

Table S5 The three vectors defining the PCRO with mid-points ¼,¼,0 in space group *Cmcm*

		Cartesian components			Nearest pseudocubic axes	
Stalk	PCRO vector	*X*	*Y*	*Z*	pseudocubic	orthorhombic
O b ← O a	$\boldsymbol{a}_1$	$a/2$	-*b*(2*y*(O2)-½)	-2*cz*(O2)	x_{PC}	[100]
O d ← O c	$\boldsymbol{a}_2$	-*a*(2*x*(O1)-½)	$b/2$	0	y_{PC}	[010]
O f ← O e	$\boldsymbol{a}_3$	*a*(2*x*(O3)-½)	*b*(2*y*(O3)-½)	$c/2$	z_{PC}	[001]

As there are no interdependencies between the PCRO vectors in this space group.

S2.3. Space group *Ibmm*

The positions quoted in Table S6 have been derived from the positions quoted for *Imma* in *International Tables for Crystallography* (Hahn, 1995).

Table S6 Relevant positions in Space Group *Ibmm* for perovskites

	Position	1	2	3	4
	B (4*a*)	0,0,0	0,0,½		
(0,0,0)+ (½,½,½)+	A & O1 (4*e*)	*x*,0,¼	$\bar{x}$,0,¾		
	O2 (8*g*)	¼,¼,*z*	¼,¾,$\bar{z}$ +½	¾,¾,$\bar{z}$	¾,¼,*z*+½

The reference structure is of $BaPbO_3$ refined at room temperature without considering anisotropic line broadening (Fu *et al.*, 2005). The following unit cell parameters apply: *a* = 6.06525 Å; *b* = 6.02504 Å; *c* = 8.50576 Å. Atomic parameters for Pb and O are as follows: Pb: 4*a*; O: 4*e*: *x*(O1) = 0.0502; 8*g* : *z*(O2) = -0.0271. Fixed oxygen coordinates are as follows: *y*(O1) = 0; *z*(O1) = ¼; *x*(O2) = ¼; *y*(O2) = ¼.

The octahedral cage coordinating the B-ion at 0,0,0 in the unit cell is taken, with the other three octahedra in the cell generated by symmetry. Pseudocubic axes are set as follows: origin of coordinates at [0,0,0]; x_{PC}: || [1$\bar{1}$0]; y_{PC}: || [110]; z_{PC}: || [001]. Analytical fractional coordinates are calculated in Table S7.

Table S7 Calculation of analytical fractional coordinates of the six octahedral anions coordinating the lead ion located at 0,0,0

Atom	Cartesian coordinates			Numerical fractional coordinates			Analytical fractional coordinates		
	X	*Y*	*Z*	*x*	*y*	*z*	*x*	*y*	*z*
Pb	0	0	0	0	0	0	0	0	0
O a (8*g*)	-1.5163	1.5063	0.2386	-¼	¼	0.0271	-¼	¼	-*z*(O2)
O b (8*g*)	1.5163	-1.5063	-0.2386	¼	-¼	-0.0271	¼	-¼	*z*(O2)
O c (8*g*)	-1.5163	-1.5063	0.2386	-¼	-¼	0.0271	-¼	-¼	-*z*(O2)
O d (8*g*)	1.5163	1.5063	-0.2386	¼	¼	-0.0271	¼	¼	*z*(O2)
O e (4*e*)	-0.3033	0.0000	-2.2014	-0.0502	0	-¼	-*x*(O1)	0	-¼
O f (4*e*)	0.3033	0.0000	2.2014	0.0502	0	¼	*x*(O1)	0	¼

The three vectors for the PCRO are now formed in Table S8 by taking the differences in the analytical fractional coordinates given in Table S7 and forming Cartesian vectors by multiplying the vectors in fractional coordinates by the orthogonalization matrix $\begin{pmatrix} a & 0 & 0 \\ 0 & b & 0 \\ 0 & 0 & c \end{pmatrix}$. The three stalk vectors so formed are parallel to the edges of the PCRO.

Table S8 The three vectors defining the PCRO with mid-points ½,0,0 in space group *Ibmm*

		Cartesian components			Nearest pseudocubic axes	
Stalk	PCRO vector	x_C	y_C	z_C	pseudocubic	orthorhombic
O b ← O a	$\boldsymbol{a}_1$	$^a/_2$	$-^b/_2$	2*cz*(O2)	x_{PC}	$[1\bar{1}0]$
O d ← O c	$\boldsymbol{a}_2$	$^a/_2$	$^b/_2$	2*cz*(O2)	x_{PC}	[110]
O f ← O e	$\boldsymbol{a}_3$	2*ax*(O1)	0	$^c/_2$	z_{PC}	[001]

The colour-coding signifies that vectors $\boldsymbol{a}_1$ and $\boldsymbol{a}_2$ are interdependent, in this case of equal length. Since $\boldsymbol{a}_1 \cdot \boldsymbol{a}_3 = \boldsymbol{a}_2 \cdot \boldsymbol{a}_3$, it follows that angles θ_{31} and θ_{23} will also be equal.

S2.4. Space group *P4/mbm*

The reference structure is of $NaNbO_3$ at 888 K (Darlington & Knight, 1999) ; ICSD 280100). The following unit cell parameters apply: a = 5.56896 Å; c = 3.94408 Å. Atomic parameters for Nb and O are as follows: Nb: 2*a*; O1: 2*b*; O2: 4*g* : *X*(O2) = 0.2281; *x*(O2)=½-X(O2); *y*(O2)=X(O2). Fixed oxygen coordinates are as follows: *x*(O1)=0; *y*(O1)=0; *z*(O1)=½; *z*(O2)=0.

The octahedral cage coordinating the B-ion at 0,0,0 in the unit cell is taken, with the other octahedron in the cell generated by symmetry. Pseudocubic axes are set as follows: origin of coordinates at [0,0,0]; x_{PC}: || [110]; y_{PC}: || [$\bar{1}$10]; z_{PC}: || [001]. Analytical fractional coordinates are calculated in Table S9.

Table S9 Calculation of analytical fractional coordinates of the six octahedral anions coordinating the titanium ion located at 0,0,0

Atom	Cartesian coordinates			Numerical fractional coordinates			Analytical fractional coordinates		
	X	*Y*	*Z*	*x*	*y*	*z*	*x*	*y*	*z*
Nb	0.0000	0.0000	0.0000	0.0000	0.0000	0.0000	0	0	0
O a (4*g*)	-1.2703	1.5142	0.0000	-0.2281	0.2719	0.0000	-*X*(O2)	-*X*(O2)+½	0
O b (4*g*)	1.2703	-1.5142	0.0000	0.2281	-0.2719	0.0000	*X*(O2)	*X*(O2)-½	0
O c (4*g*)	-1.5142	-1.2703	0.0000	-0.2719	-0.2281	0.0000	*X*(O2)-½	-*X*(O2)	0
O d (4*g*)	1.5142	1.2703	0.0000	0.2719	0.2281	0.0000	-*X*(O2)+½	*X*(O2)	0
O e (2*b*)	0.0000	0.0000	-1.9720	0.0000	0.0000	-0.5000	0	0	-½
O f (2*b*)	0.0000	0.0000	1.9720	0.000	0.0000	0.5000	0	0	½

The three vectors for the PCRO are now formed in Table S10 by taking the differences in the analytical fractional coordinates given in Table S9 and forming Cartesian vectors by multiplying the vectors in fractional coordinates by the orthogonalization matrix $\begin{pmatrix} a & 0 & 0 \\ 0 & a & 0 \\ 0 & 0 & c \end{pmatrix}$. The three stalk vectors so formed are parallel to the edges of the PCRO.

Table S10 The three vectors defining the PCRO with mid-points 0,0,0 in space group *P4/mbm*

		Cartesian components			Nearest pseudocubic axes	
Stalk	PCRO vector	*X*	*Y*	*Z*	pseudocubic	tetragonal
O b ← O a	$\boldsymbol{a}_1$	2*aX*(O2)	*a*(2*X*(O2)-1)	0	x_{PC}	[1$\bar{1}$0]
O d ← O c	$\boldsymbol{a}_2$	-*a*(2*X*(O2)-1)	2*aX*(O2)	0	y_{PC}	[110]
O f ← O e	$\boldsymbol{a}_3$	0	0	*c*	z_{PC}	[001]

The scalar products between all three pairs of vectors are zero. Therefore all angles will be equal to 90°. Furthermore, vectors $\boldsymbol{a}_1$ and $\boldsymbol{a}_2$ are interdependent and of equal length.

S2.5. Space group *P4₂/nmc*

The reference structure is of $CaMnO_3$ (Zagorac *et al.* (2014); ICSD 670342). The following unit cell parameters apply: *a* = 7.59 Å; *c* = 7.3 Å. Atomic parameters for Mn and O are as follows (Origin Choice 2): Mn: 8*e*; O1: 8*f*: *x*(O1) = 0.0268; O2: 8*g* : *y*(O2) = 0.0411, *z*(O2) = 0.9726; O3: 8*g*: *y*(O3) = 0.964, *z*(O3) = 0.5325.

The octahedral cage coordinating the B-ion at 0,0,0 in the unit cell is taken, with the other seven octahedra in the cell generated by symmetry. Pseudocubic axes are set as follows: origin of coordinates at [0,0,0]; x_{PC}: || [100]; y_{PC}: || [010]; z_{PC}: || [001]. Analytical fractional coordinates are calculated in Table S11.

Table S11 Calculation of analytical fractional coordinates of the six octahedral anions coordinating the manganese ion located at 0,0,0

Atom	Cartesian coordinates			Numerical fractional coordinates			Analytical fractional coordinates		
	X	*Y*	*Z*	*x*	*y*	*z*	*x*	*y*	*z*
Mn	0.0000	0.0000	0.0000	0.0000	0.0000	0.0000	0	0	0
O a (8*g*)	-1.8975	-0.3119	0.2000	-0.2500	-0.0411	0.0274	-¼	-*y*(O2)	-*z*(O2)+1
O b (8*g*)	1.8975	0.3119	-0.2000	0.2500	0.0411	-0.0274	¼	*y*(O2)	*z*(O2)-1
O c (8*g*)	0.2732	-1.8975	-0.2372	0.0360	-0.2500	-0.0325	-*y*(O3)+1	-¼	-*z*(O3)+½

O d (8*g*)	-0.2732	1.8975	0.2372	-0.0360	0.2500	0.0325	*y*(O3)-1	¼	*z*(O3)-½
O e (8*f*)	-0.2034	0.2034	-1.8250	-0.0268	0.0268	-0.2500	-*x*(O1)	*x*(O1)	-¼
O f (8*f*)	0.2034	-0.2034	1.8250	0.0268	-0.0268	0.2500	*x*(O1)	-*x*(O1)	¼

The three vectors for the PCRO are now formed in Table S12 by taking the differences in the analytical fractional coordinates given in Table S11 and forming Cartesian vectors by multiplying the vectors in fractional coordinates by the orthogonalization matrix $\begin{pmatrix} a & 0 & 0 \\ 0 & b & 0 \\ 0 & 0 & c \end{pmatrix}$. The three stalk vectors so formed are parallel to the edges of the PCRO.

Table S12 The three vectors defining the PCRO with mid-point 0,0,0 in space group *P*4$_2$*/nmc*

		Cartesian components			Nearest pseudocubic axes	
Stalk	PCRO vector	*X*	*Y*	*Z*	pseudocubic	tetragonal
O b ← O a	$\boldsymbol{a}_1$	$^a/_2$	2*ay*(O2)	2*c*(*z*(O2)-1)	x_{PC}	[100]
O d ← O c	$\boldsymbol{a}_2$	2*a*(*y*(O3)-1)	$^a/_2$	*c*(2*z*(O3)-1)	y_{PC}	[010]
O f ← O e	$\boldsymbol{a}_3$	2*ax*(O1)	-2*ax*(O1)	$^c/_2$	z_{PC}	[001]

There are no interdependent PCRO vectors in this space group.

S2.6. Space group *I4/mcm*

The reference structure is of $CaTiO_3$ at 1523 K (Yashima & Ali (2009) ; ICSD 162919). The following unit cell parameters apply: *a* = 5.4841 Å; *c* = 7.765 Å. Atomic parameters for Ti and O are as follows: Ti: 4*c*; O1: 4*a*; O2: 8*h* : *x*(O2) = 0.2165; *y*(O2) = *x*(O2) + ½. Fixed oxygen coordinates are as follows: *z*(O2) = 0; *x*(O1) = 0; *y*(O1) = 0; *z*(O1) = ¼.

The octahedral cage coordinating the B-ion at 0,0,0 in the unit cell is taken, with the other three octahedra in the cell generated by symmetry. Pseudocubic axes are set as follows: origin of coordinates at [0,0,0]; x_{PC}: ‖ [1$\bar{1}$0]; y_{PC}: ‖ [110]; z_{PC}: ‖ [001]. Analytical fractional coordinates are calculated in Table S13.

Table S13 Calculation of analytical fractional coordinates of the six octahedral anions coordinating the titanium ion located at 0,0,0

Atom	Cartesian coordinates			Numerical fractional coordinates			Analytical fractional coordinates		
	X	*Y*	*Z*	*x*	*y*	*z*	*x*	*y*	*z*
Ti	0.0000	0.0000	0.0000	0.0000	0.0000	0.0000	0	0	0
O a (8*h*)	-1.1873	1.5547	0.0000	-0.2165	0.2835	0.0000	-*x*(O2)	-*x*(O2)+½	0
O b (8*h*)	1.1873	-1.5547	0.0000	0.2165	-0.2835	0.0000	*x*(O2)	*x*(O2)-½	0
O c (8*h*)	-1.5547	-1.1873	0.0000	-0.2835	-0.2165	0.0000	*x*(O2)-½	-*x*(O2)	0
O d (8*h*)	1.5547	1.1873	0.0000	0.2835	0.2165	0.0000	-*x*(O2)+½	*x*(O2)	0
O e (4*a*)	0.0000	0.0000	-1.9413	0.0000	0.0000	-0.2500	0	0	-¼
O f (4*a*)	0.0000	0.0000	1.9413	0.000	0.0000	0.2500	0	0	¼

The three vectors for the PCRO are now formed in Table S14 by taking the differences in the analytical fractional coordinates given in Table S13 and forming Cartesian vectors by multiplying the vectors in fractional coordinates by the orthogonalization matrix $\begin{pmatrix} a & 0 & 0 \\ 0 & a & 0 \\ 0 & 0 & c \end{pmatrix}$. The three stalk vectors so formed are parallel to the edges of the PCRO.

Table S14 The three vectors defining the PCRO with mid-points 0,0,0 in space group *I*4/*mcm*

		Cartesian components			Nearest pseudocubic axes	
Stalk	PCRO vector	*X*	*Y*	*Z*	pseudocubic	tetragonal
O b ← O a	$\boldsymbol{a}_1$	2*ax*(O2)	*a*(2*x*(O2)-1)	0	x_{PC}	$[1\bar{1}0]$
O d ← O c	$\boldsymbol{a}_2$	- *a*(2*x*(O2)-1)	2*ax*(O2)	0	y_{PC}	[110]
O f ← O e	$\boldsymbol{a}_3$	0	0	$^c/_2$	z_{PC}	[001]

The scalar products between all three pairs of vectors are zero. Therefore all angles will be equal to 90°. Furthermore, vectors $\boldsymbol{a}_1$ and $\boldsymbol{a}_2$ are interdependent and of equal length. It is to be noted that the entries in

Table S14 are identical to those in Table S10, apart from the change from *c* to *c*/2 in the entry for the *z*-component of vector $\boldsymbol{a}_3$. This is connected with the change in tilt-system from $a^0a^0c^+$ to $a^0a^0c^-$.

S2.7. Space group $R\bar{3}c$

The reference structure is of $La(Cr_{0.2}Ni_{0.8})O_3$ at 298 K (Yang, 2008) ; ICSD 173475). The following unit cell parameters, referred to hexagonal axes, apply: *a* = 5.4809 Å; *c* = 13.2070 Å. Atomic parameters for Cr/Ni and O are as follows: Cr/Ni: 6*b*; O: 18*e*: *x*(O)=0.5517. Fixed oxygen coordinates are as follows: *y*(O)=0; *z*(O) = ¼.

The octahedral cage coordinating the B-ion at 0,0,0 in the unit cell is taken, with the other five octahedra in the cell generated by symmetry. Pseudocubic axes are set as follows: origin of coordinates at [0,0,0]; x_{PC}: $\frac{1}{3}[241]$; y_{PC}: $\frac{1}{3}[\bar{4}\,\bar{2}1]$; z_{PC}: $\frac{1}{3}[2\bar{2}1]$. Analytical fractional coordinates are calculated in Table S15.

Table S15 Calculation of analytical fractional coordinates of the six octahedral anions coordinating the B-ion located at 0,0,0

Atom	Cartesian coordinates			Numerical fractional coordinates			Analytical fractional coordinates		
	X	*Y*	*Z*	*x*	*y*	*z*	*x*	*y*	*z*
Cr/Ni	0.0000	0.0000	0.0000	0.0000	0.0000	0.0000	0	0	0
O a (18 *e*)	-0.5457	-1.5119	1.1006	-0.1150	-0.3333	-0.0833	$x(O)-\frac{2}{3}$	-1/3	-1/12
O b (18 *e*)	0.5457	1.5119	-1.1006	0.1150	0.3333	0.0833	$-x(O)+\frac{2}{3}$	1/3	1/12
O c (18 *e*)	1.5822	0.2834	-1.1006	0.3333	0.2184	-0.0833	1/3	$x(O)-\frac{1}{3}$	-1/12
O d (18 *e*)	-1.5822	-0.2834	1.1006	-0.3333	-0.2184	0.0833	-1/3	$-x(O)+\frac{1}{3}$	1/12
O e (18 *e*)	-1.0365	1.2285	-1.1006	-0.2184	0.1150	-0.0833	$-x(O)+\frac{1}{3}$	$-x(O)+\frac{2}{3}$	-1/12
O f (18 *e*)	1.0365	-1.2285	1.1006	0.2184	-0.1150	0.0833	$x(O)-\frac{1}{3}$	$x(O)-\frac{2}{3}$	1/12

The three vectors for the PCRO are now formed in Table S16 by taking the differences in the analytical fractional coordinates given in Table S15 and forming Cartesian vectors by multiplying the vectors in

fractional coordinates by the orthogonalization matrix $\begin{pmatrix} \sqrt{3}a/2 & 0 & 0 \\ -a/2 & a & 0 \\ 0 & 0 & c \end{pmatrix}$. The three stalk vectors so formed are parallel to the edges of the PCRO.

Table S16 The three vectors defining the PCRO with mid-point 0,0,0 in space group $R\bar{3}c$

		Cartesian components			Nearest pseudocubic axes	
Stalk	PCRO vector	X	Y	Z	pseudocubic	hexagonal
O b ← O a	$\boldsymbol{a}_1$	$\sqrt{3}a(-x(\mathrm{O})+2/3)$	$ax(\mathrm{O})$	$\frac{c}{6}$	x_{PC}	$\frac{1}{3}[241]$
O d ← O c	$\boldsymbol{a}_2$	$-a/\sqrt{3}$	$-a(2x(\mathrm{O})-1)$	$\frac{c}{6}$	y_{PC}	$\frac{1}{3}[\bar{4}\bar{2}1]$
O f ← O e	$\boldsymbol{a}_3$	$\sqrt{3}a(x(\mathrm{O})-1/3)$	$a(x(\mathrm{O})-1)$	$\frac{c}{6}$	z_{PC}	$\frac{1}{3}[2\bar{2}1]$

All vectors are interdependent and of equal length. Furthermore, the angles between them are equal.

S2.8. Space group $Pm\bar{3}m$

The reference structure is of lueshite ($NaNbO_3$) at 923 K (Mitchell *et al.* (2014); ICSD 192407). The following unit cell parameters apply: a = 3.94018 Å. Atomic parameters for Nb and O are as follows: Nb: 1*a*; O: 3*d*. Fixed oxygen coordinates are as follows: x(O) = ½; y(O) = 0; z(O) = 0.

The octahedral cage coordinating the B-ion at 0,0,0 in the unit cell is taken. Pseudocubic axes are equal to the cubic unit cell axes, as there is no octahedral tilting in the aristotype. Analytical coordinates for the six octahedral vertices are as follows: -½,0,0; ½,0,0; 0,-½,0; 0,½,0; 0,0-½; 0,0,½. These give rise to three stalk vectors in Table S17, which are parallel to the edges of the PCRO. In this case, the PCRO is a perfect cube with the form of the unit cell.

Table S17 The three vectors defining the PCRO in the $Pm\bar{3}m$ aristotype

		Cartesian components			Nearest pseudocubic axes	
Stalk	PCRO vector	X	Y	Z	pseudocubic	cubic
O b ← O a	$\boldsymbol{a}_1$	a	0	0	x_C	[100]

O d ← O c	$\boldsymbol{a}_2$	0	a	0	y_C	[010]
O f ← O e	$\boldsymbol{a}_3$	0	0	a	z_C	[001]

All three vectors are interdependent, of equal length and perpendicular to one another.

S3. Algorithms used for calculating tilt angles

S3.1. Space group *Pbnm* and other non-rhombohedral space groups

In Fig. 6, the octahedron at [0,0.5] is highlighted for analysis. In the **Pbmn(4b)** table in the accompanying EXCEL datafile, the following Cartesian coordinates apply to the PCRO axes, *i.e.* octahedral stalks and pseudocubic axes.

PCRO	0½0			Nearest pseudocubic axis		
	X (Å)	*Y* (Å)	*Z* (Å)	*X* (Å)	*Y* (Å)	*Z* (Å)
$\boldsymbol{a}_1$	2.2697	-3.1385	-0.5720	5.3709	-5.4280	0.0000
$\boldsymbol{a}_2$	3.1012	2.2895	-0.5720	5.3709	5.4280	0.0000
$\boldsymbol{a}_3$	0.7605	-0.1846	3.8134	0.0000	0.0000	7.6268

The first two lines with a green background correspond to the x_{PC} and y_{PC} axes respectively, about which the a^- tilting occurs. The projections O c → O c' and O d → O d' may be made by a coordinate transformation, after which the new X' and Y' axes lie in the plane of projection, with Z'|| x_{PC}. Similarly, projections O a → O a' and O b → O b' may be made by a transformation after which the new X' and Y' axes lie in the plane of projection, with Z'|| y_{PC}. The general form of a 3x3-matrix to carry out a rotation of θ about an axis with unit vector [a_X, a_Y, a_Z] is as follows (Williams,1971): $\begin{pmatrix} a_X^2C + A & a_Xa_YC - a_ZS & a_Xa_ZC + a_YS \\ a_Xa_YC + a_ZS & a_Y^2C + A & a_Ya_ZC - a_XS \\ a_Xa_ZC - a_YS & a_Ya_ZC + a_XS & a_Z^2C + A \end{pmatrix}$, with $A = \cos(\theta)$, $C = 1 - A$ and $S = \sin(\theta)$. (S3.1). The rotation axes for the two transformations are given by the vector products $\boldsymbol{x}_{PC} \times [001]$ and $\boldsymbol{y}_{PC} \times [001]$ followed by normalization. In both cases, the rotation angle is 90°, leading to $A = 0, C = 1$ and $S = 1$. The matrix of (S3.1) is accordingly reduced to the form

$$\begin{pmatrix} a_X^2 & a_Xa_Y - a_Z & a_Xa_Z + a_Y \\ a_Xa_Y + a_Z & a_Y^2 & a_Ya_Z - a_X \\ a_Xa_Z - a_Y & a_Ya_Z + a_X & a_Z^2 \end{pmatrix}$$

(S3.2). The numerical results obtained with the above data in EXCEL table **Pbmn(4b)** are as follows:

	AA	AB	AC	AD	AE	AF	AG	AH	AI	AJ	AK	AL	AM	AN
1	e($\mathbf{a}_{PC}$)	rot. axis	rotmat ⊥ $\boldsymbol{a}_{1,XY}$			e'($\mathbf{a}_{PC}$)	z	z'	e($\mathbf{a}_2$)	e'($\mathbf{a}_2$)	∠(z')	∠($\mathbf{a}_2$')	∠($\mathbf{a}_2$')-∠(z')	$∠_{tilt}$($\mathbf{a}_2$)
2	0.7034	-0.7108	0.5053	0.5000	-0.7034	0.0000	0	-0.7034	0.7958	0.7991	134.70	36.17	-98.53	-8.53
3	-0.7108	-0.7034	0.5000	0.4947	0.7108	0.0000	0	0.7108	0.5875	0.5842				
4	0.0000	0.0000	0.7034	-0.7108	0.0000	1.0000	1	0.0000	-0.1468	0.1421				
5	e($\mathbf{b}_{PC}$)	rot. axis	rotmat ⊥ $\boldsymbol{a}_{2,XY}$			e'($\mathbf{b}_{PC}$)	z	z'	e($\mathbf{a}_1$)	e'($\mathbf{a}_1$)	∠(z')	∠($\mathbf{a}_1$')	∠($\mathbf{a}_1$')-∠(z')	$∠_{tilt}$($\mathbf{a}_1$)
6	0.7034	0.7108	0.5053	-0.5000	-0.7034	0.0000	0	-0.7034	0.5797	0.7965	-134.70	-36.18	98.51	8.51
7	0.7108	-0.7034	-0.5000	0.4947	-0.7108	0.0000	0	-0.7108	-0.8016	-0.5826				
8	0.0000	0.0000	0.7034	0.7108	0.0000	1.0000	1	0.0000	-0.1461	-0.1621				

$\mathbf{e}(\mathbf{a}_{\mathrm{PC}})$ and $\mathbf{e}(\mathbf{b}_{\mathrm{PC}})$ are (in column AA) are unit vectors for the x_{PC} and y_{PC} axes, leading to the rotation axes in column AB. The two rotation matrices are in columns AC-AE. Column AF gives the results of multiplying $\mathbf{e}(\mathbf{a}_{\mathrm{PC}})$ and $\mathbf{e}(\mathbf{b}_{\mathrm{PC}})$ by the rotation matrices. It provides confirmation that the x_{PC} and y_{PC} axes are realigned ||Z' by the coordinate transformation. After the coordinate transformations, the orthorhombic z-axis lies in the $X'Y'$ plane with unit vectors in column AH (yellow background). Column AI contains the unit vectors for octahedral stalk vectors $\boldsymbol{a}_2$ and $\boldsymbol{a}_1$. The projections of these two vectors in the $X'Y'$ plane are given in cells AJ2:AJ3 and AJ6:AJ7. Use of the EXCEL ATAN2 function yields, in columns AK and AL, the angles between -180° and +180° for vector z' (in column AH) and the vectors in column AJ. The difference between these two angles, which is the angle between the orthorhombic z-axis and the projection of the relevant stalk vector, is in column AM. The tilt angle, i.e. the angle between projected stalk vector and xy-plane, is given by subtracting or adding 90° to the angle in column AM. The two tilt angles are contained in cells AN2 and AN6.

The out-of-phase tilting (a^{-}) may be verified by deriving the PCRO vectors of a second octahedron with B-ion at [x,y] = [0.5,0.0] (Fig. 6). The corresponding results are given in the following two tables.

PCRO	0½0			Nearest pseudocubic axis		
	X (Å)	Y (Å)	Z (Å)	X (Å)	Y (Å)	Z (Å)
$\boldsymbol{a}_1$	3.1012	-2.2895	0.5720	5.3709	-5.4280	0.0000
$\boldsymbol{a}_2$	2.2697	3.1385	0.5720	5.3709	5.4280	0.0000
$\boldsymbol{a}_3$	-0.7605	-0.1846	3.8134	0.0000	0.0000	7.6268

It is observed that the PCRO vectors (with blue background) are different for this octahedron, whereas the pseudocubic axes (with green background) are unaltered.

	AA	AB	AC	AD	AE	AF	AG	AH	AI	AJ	AK	AL	AM	AN
1	e($\mathbf{a}_{PC}$)	rot. axis	rotmat ⊥ $\boldsymbol{a}_{1,XY}$			e'($\mathbf{a}_{PC}$)	z	z'	e($\mathbf{a}_2$)	e'($\mathbf{a}_2$)	∠(z')	∠($\mathbf{a}_2$')	∠($\mathbf{a}_2$')-∠(z')	$∠_{tilt}$($\mathbf{a}_2$)
2	0.7034	-0.7108	0.5053	0.5000	-0.7034	0.0000	0	-0.7034	0.5797	0.5909	134.70	53.21	-81.49	8.51
3	-0.7108	-0.7034	0.5000	0.4947	0.7108	0.0000	0	0.7108	0.8016	0.7903				
4	0.0000	0.0000	0.7034	-0.7108	0.0000	1.0000	1	0.0000	0.1461	-0.1621				
5	e($\mathbf{b}_{PC}$)	rot. axis	rotmat ⊥ $\boldsymbol{a}_{2,XY}$			e'($\mathbf{b}_{PC}$)	z	z'	e($\mathbf{a}_1$)	e'($\mathbf{a}_1$)	∠(z')	∠($\mathbf{a}_1$')	∠($\mathbf{a}_1$')-∠(z')	$∠_{tilt}$($\mathbf{a}_1$)
6	0.7034	0.7108	0.5053	-0.5000	-0.7034	0.0000	0	-0.7034	0.7958	0.5926	-134.70	-53.22	81.47	-8.53
7	0.7108	-0.7034	-0.5000	0.4947	-0.7108	0.0000	0	-0.7108	-0.5875	-0.7929				
8	0.0000	0.0000	0.7034	0.7108	0.0000	1.0000	1	0.0000	0.1468	0.1421				

The $\boldsymbol{a}_2$ stalk has a tilt angle of +8.51°, compared to -8.53° for the first octahedron at [x,y] = [0.0,0.5]. Similarly, the $\boldsymbol{a}_1$ stalk has a tilt angle of -8.53°, compared to +8.51° for the first octahedron at [x,y] = [0.0,0.5]. The tilting around the x_{PC} axis is therefore equivalent to the tilting around the y_{PC} axis, leading to the notation a^{-} for both axes. The tilt angle of the system is given by (8.53-(-8.51))/2 = 8.52°.

Calculation of the in-phase c^{+}tilt angle is more straightforward. Projections of the stalk vectors $\boldsymbol{a}_1$and $\boldsymbol{a}_2$ in the xy-plane are formed by setting $a_{1z} = 0$ and $a_{2z} = 0$.

For the octahedron at [0,0.5]: $[a_{1X}, a_{1Y}]$ = [2.2697, -3.1385] and $[a_{2X}, a_{2Y}]$ = [3.1012, 2.2895].

For the octahedron at [0.5,0]: $[a_{1X}, a_{1Y}]$ = [3.1012, -2.2895] and $[a_{2X}, a_{2Y}]$ = [2.2697, 3.1385].

The nearest pseudocubic axes remain constant as follows: x_{PC}: $[X, Y]$ = [5.3709, -5.4280]; y_{PC}: $[X, Y]$ = [5.3709, 5.4280]. Scalar products of these vectors yield the cosines of the tilt angles, with the following results: Octahedron at [0,0.5]: $\phi_{c,1}$ = 8.82°; $\phi_{c,2}$ = 8.87°; Octahedron at [0.5,0]: $\phi_{c,1}$ = 8.87°; $\phi_{c,2}$ = 8.82°. The mean value of 8.84° is taken as the tilt angle.

These algorithms for space group *Pbnm* may be used without modification for all the other non-rhombohedral space groups, since the z_{PC}-axis is perpendicular to the $x_{PC}y_{PC}$-plane. They are implemented in all the tables in the EXCEL file contained in the supporting information.

Since the none of the pseudocubic axes in the rhombohedral space group $R\bar{3}c$ are perpendicular to one another, a special algorithm is required, as summarised in the following subsection.

S3.2. Space group $R\bar{3}c$

The pseudocubic axes are equivalent to the unit cell axes in the rhombohedral setting of the space group. In order to calculate the a^- tilt angle, which is the same for all three axes, two neighbouring BO_6 octahedra along one pseudocubic axis are analysed, the first at [0,0,0] and the second half-way along this axis at $\frac{1}{6}[241] = \left[\frac{1}{3}\frac{2}{3}\frac{1}{6}\right]$. The tilt angle is determined by the projections of the oxygen ions lying approximately transverse to the axis in its perpendicular plane. For the octahedron at [0,0,0], the oxygen coordinates are atoms O c, O d, O e and O f, which were quoted in Table S15. This information is reproduced in Table S18, along with the corresponding coordinates of transverse atoms O c', O d', O e' and O f' that belong to the octahedron with B-ion at $\left[\frac{1}{3}\frac{2}{3}\frac{1}{6}\right]$.

Table S18 Calculation of analytical fractional coordinates of the four octahedral oxygen ions lying approximately transverse to axis $\frac{1}{3}[241]$ in the octahedra with B-ions located at [0,0,0] and $\left[\frac{1}{3}\frac{2}{3}\frac{1}{6}\right]$.

Atom	Cartesian coordinates			Numerical fractional coordinates			Analytical fractional coordinates		
[0,0,0]	*X*	*Y*	*Z*	*x*	*y*	*z*	*x*	*y*	*z*
Cr/Ni	0.0000	0.0000	0.0000	0.0000	0.0000	0.0000	0	0	0
O c	1.5822	0.2834	-1.1006	0.3333	0.2184	-0.0833	1/3	x(O)-$\frac{1}{3}$	-1/12
O d	-1.5822	-0.2834	1.1006	-0.3333	-0.2184	0.0833	-1/3	-x(O)+$\frac{1}{3}$	1/12
O e	-1.0365	1.2285	-1.1006	-0.2184	0.1150	-0.0833	-x(O)+$\frac{1}{3}$	-x(O)+$\frac{2}{3}$	-1/12

O f	1.0365	-1.2285	1.1006	0.2184	-0.1150	0.0833	x(O)-$\frac{1}{3}$	x(O)-$\frac{2}{3}$	1/12
$\left[\frac{1}{3}\frac{2}{3}\frac{1}{6}\right]$	*X*	*Y*	*Z*	*x*	*y*	*z*	*x*	*y*	*z*
Cr/Ni	1.5822	2.7405	2.2012	0.3333	0.6667	0.1667	1/3	2/3	1/6
O c’	3.1644	2.4571	1.1006	0.6667	0.7816	0.0833	2/3	-x(O)+$\frac{4}{3}$	1/12
O d’	0.0000	3.0238	3.3018	0.0000	0.5517	0.2500	0	x(O)	1/4
O e’	1.0365	4.2524	1.1006	0.2184	0.8850	0.0833	x(O)-$\frac{1}{3}$	x(O)+$\frac{1}{3}$	1/12
O f’	2.1279	1.2285	3.3018	0.4483	0.4483	0.2500	-x(O)+1	-x(O)+1	1/4

The method of calculation of the tilt angle may be inspected in the **R-3c** sheet of the EXCEL file in the supporting information, from which the following tables are taken.

	AA	AB	AC	AD	AE	AF	AG	AH	AI	AJ	AK
1		B[0,0,0]	O c	O d	O e	O f	B[0,0,0]	O c	O d	O e	O f
2	*x*	0,0000	0,3333	-0,3333	-0,2184	0,2184	0,0000	1,5822	-1,5822	-1,0365	1,0365
3	*y*	0,0000	0,2184	-0,2184	0,1150	-0,1150	0,0000	0,2834	-0,2834	1,2285	-1,2285
4	*z*	0,0000	-0,0833	0,0833	-0,0833	0,0833	0,0000	-1,1006	1,1006	-1,1006	1,1006
5		B[1/3,2/3,1/6]	O c'	O d'	O e'	O f'	B[1/3,2/3,1/6]	O c'	O d'	O e'	O f'
6	*x*	0,3333	0,6667	0,0000	0,2184	0,4483	1,5822	3,1644	0,0000	1,0365	2,1279
7	*y*	0,6667	0,7816	0,5517	0,8850	0,4483	2,7405	2,4571	3,0238	4,2524	1,2285
8	*z*	0,1667	0,0833	0,2500	0,0833	0,2500	2,2012	1,1006	3,3018	1,1006	3,3018
9		Orthogonalization matrix:									
10		4,7466	0,0000	0,0000							
11		-2,7405	5,4809	0,0000							
12		0,0000	0,0000	13,2070							

Columns AC to AF contain the fractional coordinates of the relevant oxygen ions, which are derived from x(O) parameter in cell N5. The corresponding Cartesian coordinates are generated in columns AH to AK by applying the orthogonalization matrix in block AB10:AD12. This is calculated from the unit cell parameters in cells K3 and K4.

	AL	AM	AN	AO	AP	AQ	AR	AS	AT
1	e(a_{PC})	rot. ax.	e(rot. ax.)	∠(z -a_{PC})			rotmat ⊥ $\boldsymbol{a}_{1,XY}$		
2	0.4105	0.7109	0.8660	55.1774	A:	0.5710	0.8928	-0.1857	-0.4105
3	0.7109	-0.4105	-0.5000		C:	0.4290	-0.1857	0.6783	-0.7109
4	0.5710	0.0000	0.0000		S:	0.8209	0.4105	0.7109	0.5710
5	7.7094	0.8209							

The vector in AL2:AL4 is the unit vector derived from the a_{PC} pseudocubic axis, around which the antiphase tilting occurs.

The rotation axis in AM2:AM4 is the vector product of **e**(**a**$_{PC}$) and [0,0,1], which generates the unit vector in AN2:AN4. The angle in cell AO2 is the angle between [0,0,1] and **e**(**a**$_{PC}$): rotation by this angle around the rotation axis causes the **e**(**a**$_{PC}$) vector to be oriented ||[0,0,1], *i.e.* ||*z*' in the new coordinate system. The rotation matrix in block AR2:AT4 performs this coordinate transformation, which is defined by matrix (S3.1) in §S3.1 When this matrix operates on Cartesian coordinates of the B ions and oxygen coordinates O c to O f and O c' to O f', the transformed x' and y' coordinates yield the relative angles of rotation of the two sets of four B-O vectors about the **a**$_{PC}$ axis. The two B ions lie on the pseudocubic axis. The EXCEL ATAN2 function is used to yield four angles for the first octahedron in cells AZ2:BC2 and four angles for the second octahedron in cells AZ6:BC6 (see following table). The differences between these two sets of angles, which are all equal to 11.81°, are contained in cells AZ8:BC8.

	AU	AV	AW	AX	AY	AZ	BA	BB	BC	BD
1	B (R)	O c(R)	O d(R)	O e(R)	O f(R)	∠(B-O c)	∠(B-O d)	∠(B-O e)	∠(B-O f)	
2	0.0000	1.8116	-1.8116	-0.7018	0.7018	20.59	-159.41	111.21	-68.79	
3	0.0000	0.6808	-0.6808	1.8083	-1.8083					
4	0.0000	0.2224	-0.2224	-0.1805	0.1805					
5	B (R)	O c'(R)	O d'(R)	O e'(R)	O f'(R)	∠(B-O c')	∠(B-O d')	∠(B-O e')	∠(B-O f')	
6	0.0000	1.9169	-1.9169	-0.3163	0.3163	8.79	-171.21	99.41	-80.59	
7	0.0000	0.2964	-0.2964	1.9093	-1.9093	Δ∠	Δ∠	Δ∠	Δ∠	Tilt angle
8	3.8547	3.6742	4.0352	4.0771	3.6323	11.81	11.81	11.81	11.81	5.90

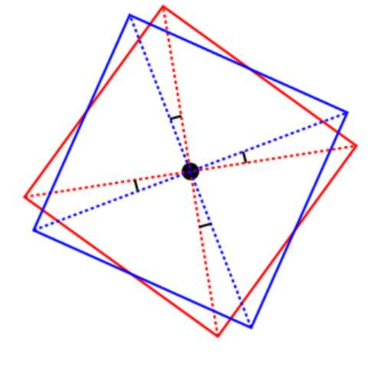

In the sketch to the right, which refers to this concrete example, the blue octahedron (O a – O b – O c – O d) has undergone an anticlockwise tilt and the red octahedron (O a' – O b' – O c' – O d') a clockwise tilt. The four arcs shown are equal to 11.81°. The effective tilt angle is taken as one half of this, *i.e.* 5.90°, since the anticlockwise and clockwise tilts add to give 11.81°

S4. Additional crystal chemical parameters for the anionic network from the AX_{12} polyhedra

The analysis leading to Table 4 of the article reveals that one or more degrees of freedom (d.o.f.) remain in all hettotypical space groups, *i.e.* $\Delta n \geq 1$, once the unit cell parameters and octahedral distortions have been taken into account. It is desirable to assign these excess d.o.f. to structural attributes of the AX_{12} polyhedra. As discussed in connection with Fig. 7 of the article, an inner X_8 coordination polyhedron exists in space group *Pbnm*. Its form may be derived from a right prism with a parallelogram base. This rationalisation also applies to all the other space groups apart from $P4_2/nmc$, $I4/mcm$ and $R\bar{3}c$. The task here is to derive the areas of the parallelogram bases from the unit cell and atomic positional parameters (Table S19).

Table S19 Derivation of cross-sectional areas of AX_8 inner polyhedra and number of tilt systems, N(tilt), for space groups *Pbnm* to *I4/mcm* and AX_6 areas in $R\bar{3}c$ with corresponding expressions for tilt-parameter η_A.

Pbnm (B in 4*b*)

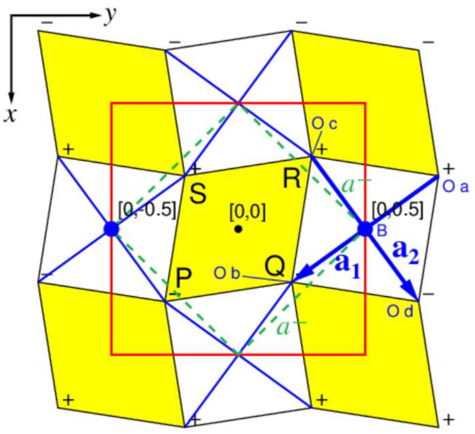

The area of parallelogram PQRS is to be determined.

Vertex P is reached by starting at the left-hand blue circle with fractional coordinates $\begin{pmatrix} 0 \\ -\frac{1}{2} \end{pmatrix}$ and proceeding along half the length of the projection of vector $\boldsymbol{a}_2$ (see Table 2 of article) in the *xy* plane. Thus it has Cartesian coordinates $\begin{pmatrix} X \\ Y \end{pmatrix} = \begin{pmatrix} 0 \\ -\frac{b}{2} \end{pmatrix} + \frac{1}{2}\begin{pmatrix} a_{2X} \\ a_{2Y} \end{pmatrix}$. Vertex Q is reached by starting at the right-hand blue circle and proceeding along half the length of the projection of vector $\boldsymbol{a}_1$. It has coordinates $\begin{pmatrix} X \\ Y \end{pmatrix} = \begin{pmatrix} 0 \\ \frac{b}{2} \end{pmatrix} + \frac{1}{2}\begin{pmatrix} a_{1X} \\ a_{1Y} \end{pmatrix}$. Vertex R has coordinates $\begin{pmatrix} X \\ Y \end{pmatrix} = \begin{pmatrix} 0 \\ \frac{b}{2} \end{pmatrix} - \frac{1}{2}\begin{pmatrix} a_{2X} \\ a_{2Y} \end{pmatrix}$. So vector **QR** = $\begin{pmatrix} -a_{2X}-a_{1X} \\ -a_{2Y}-a_{1Y} \end{pmatrix}$ and vector **QP** = $\begin{pmatrix} 0 \\ -b \end{pmatrix} + \frac{1}{2}\begin{pmatrix} a_{2X} - a_{1X} \\ a_{2Y}-a_{1Y} \end{pmatrix}$. Therefore parallelogram PQRS has area $A(PQRS) =$

$$\frac{1}{4}\begin{vmatrix} -a_{2X} - a_{1X} & -a_{2Y} - a_{1Y} \\ a_{2X} - a_{1X} & -2b + a_{2Y} - a_{1Y} \end{vmatrix} = \frac{ab}{4}(1 - [3 - 4x(O2)][4y(O2) - 1]).$$

In the absence of octahedral tilting about the *z*-axis, PQRS would be a rectangle of area $\frac{ab}{4}$. So the AX_8-related tilt ratio, $\eta_A = \frac{A(PQRS)}{ab/4} =$ $(1 - [3 - 4x(O2)][4y(O2) - 1])$. (S4.1). This is less than one and falls with increasing degree of *z*-axis tilting.

N(tilt): 2. Parallelogram PQRS formation indicates $c^{\pm}$ tilting; equal and opposite + and – displacements ∥ z point to $a^{\pm}a^{\pm}$ tilting. Alternate senses of rotation along the x_{PC} and y_{PC} axes identify this as a^-a^-. Assignation of these tilts does not require the octahedra to be regular.

Pbnm (B in 4*a*)

The origin of coordinates is now at point T, so that point S has coordinates $\frac{1}{2}\begin{pmatrix} a_{1X} \\ a_{1Y} \end{pmatrix}$ and point R coordinates $\frac{1}{2}\begin{pmatrix} a_{2X} \\ a_{2Y} \end{pmatrix}$. Point P is related by a centre-of-symmetry at $\frac{1}{2}\begin{pmatrix} a \\ 0 \end{pmatrix}$ to Point R and has coordinates $\begin{pmatrix} a \\ 0 \end{pmatrix} - \frac{1}{2}\begin{pmatrix} a_{2X} \\ a_{2Y} \end{pmatrix}$. It follows that vector **SP** = $\begin{pmatrix} a \\ 0 \end{pmatrix} - \frac{1}{2}\begin{pmatrix} a_{2X}+a_{1X} \\ a_{2Y}+a_{1Y} \end{pmatrix}$ and vector **SR** = $\frac{1}{2}\begin{pmatrix} a_{2X} - a_{1X} \\ a_{2Y}-a_{1Y} \end{pmatrix}$. Therefore parallelogram PQRS has area

$$A(PQRS) = \frac{1}{4}\begin{vmatrix} 2a - a_{2X} - a_{1X} & -a_{2Y} - a_{1Y} \\ a_{2X} - a_{1X} & a_{2Y}-a_{1Y} \end{vmatrix} =$$

$$\frac{1}{4}\begin{vmatrix} a & b(1 - 4y(O2)) \\ a(4x(O2) - 1) & b \end{vmatrix} = \frac{ab}{4}([1 + [4x(O2) - 1][4y(O2) - 1]).$$

Consequently $\eta_A = \frac{A(PQRS)}{ab/} =$ $(1 - [4x(O2) - 1][4y(O2) - 1])$. (S4.2)

Cmcm

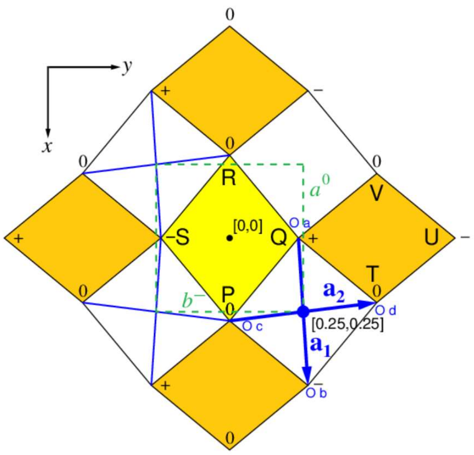

The areas of the yellow and brown parallelograms are to be determined.

Vertex P is reached by starting at the blue circle with fractional coordinates $\begin{pmatrix}1/4\\1/4\end{pmatrix}$ and proceeding along half the length of the projection of vector $-\boldsymbol{a}_2$ (see Table S5) in the *xy* plane. Thus it has coordinates $\begin{pmatrix}X\\Y\end{pmatrix} = \begin{pmatrix}\frac{a}{4}\\\frac{b}{4}\end{pmatrix} - \frac{1}{2}\begin{pmatrix}a_{2X}\\a_{2Y}\end{pmatrix}$. Vertex Q is reached from the same start-point and proceeding along half the length of the projection of vector $-\boldsymbol{a}_1$. It has coordinates $\begin{pmatrix}X\\Y\end{pmatrix} = \begin{pmatrix}\frac{a}{4}\\\frac{b}{4}\end{pmatrix} - \frac{1}{2}\begin{pmatrix}a_{1X}\\a_{1Y}\end{pmatrix}$. Vertex T is reached from the same start-point and proceeding along half the length of the projection of vector $\boldsymbol{a}_2$. It has coordinates $\begin{pmatrix}X\\Y\end{pmatrix} = \begin{pmatrix}\frac{a}{4}\\\frac{b}{4}\end{pmatrix} + \frac{1}{2}\begin{pmatrix}a_{2X}\\a_{2Y}\end{pmatrix}$. Vertex R has the same Y and Z coordinates as vertex P, with the X coordinate equal to minus the X-coordinate of point P, *i.e.* $\begin{pmatrix}X\\Y\end{pmatrix} = \begin{pmatrix}-\frac{a}{4}\\\frac{b}{4}\end{pmatrix} + \frac{1}{2}\begin{pmatrix}a_{2X}\\-a_{2Y}\end{pmatrix}$. Vertex V is similarly related to point T, *i.e.* $\begin{pmatrix}X\\Y\end{pmatrix} = \begin{pmatrix}-\frac{a}{4}\\\frac{b}{4}\end{pmatrix} + \frac{1}{2}\begin{pmatrix}-a_{2X}\\a_{2Y}\end{pmatrix}$. The parallelogram areas are calculated by means of vectors **QR**, **QP**, **QT** and **QV**. The results are as follows.

$$A(PQRS) = \frac{1}{4}(a_{1y} - a_{2y})(2a_{2x} - a) = 2abx(01)y(02) \qquad (S4.3)$$

$$A(QTUV) = \frac{1}{2}(a_{1y} + a_{2y})(2a_{2x} + a) = \frac{1}{2}ab(1 - 2x(01))(1 - 2y(02)) \qquad (S4.4)$$

The reference area for degree of *z*-axis tilting is an eighth of the area of the unit cell in the *xy*-plane, *i.e.* $\frac{ab}{8}$.
Accordingly, $\eta_{A,1} = 16x(O1)y(O2)$ (S4.5) and $\eta_{A,2} = 4\ (1 - 2x(01))(1 - 2y(02))$ (S4.6)

N(tilt): 2. Parallelogram PQRS formation indicates $c^{\pm}$ tilting. The single set of equal and opposite displacements ∥ z, only applies to rotations about the x-axis, leading to the assignation $a^{-}b^{0}$. Assignation of these tilts does not require the octahedra to be regular.

Ibmm

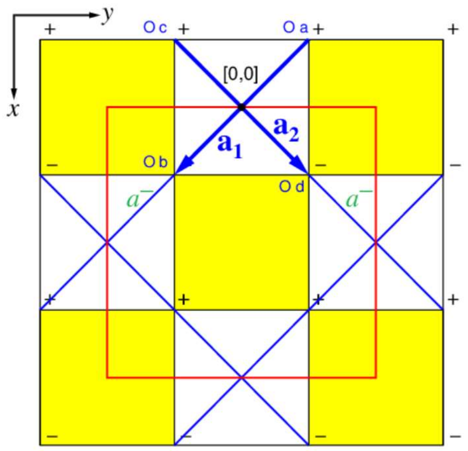

The projected areas of the octahedra and the AX_8 polyhedra in the *zx* plane are equal. They are both equal to $ab/4$. Since the reference area for degree of *y*-axis tilting is also equal to $\frac{ab}{4}$, η_A=1 (S4.7). This indicates that that there is no tilting about the *y*-axis.

N(tilt): 2. Since the upper and lower faces of the AX_8 polyhedra are rectangles in projection, there is no tilting about the *z*-axis: c^0. The single set of equal and opposite displacements ∥ z, applies to both the pseudocubic axes xC and yC, leading to the assignation a^-a^-. Assignation of these tilts does not require the octahedra to be regular.

P*4/*mbm* and *I*4/*mcm

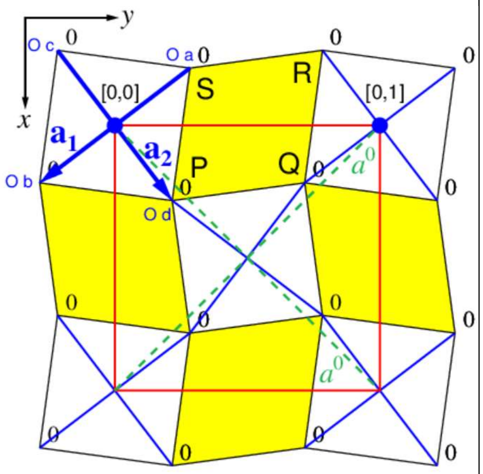

Both space groups share the same geometry in the *z* = 0 plane.

The AX_8 inner polyhedra are right prisms in *P*4/*mbm*. In space group *I*4/*mcm*, the top face is generated from face PQRS by a 2-fold screw axis. In both cases, the area of parallelogram PQRS is to be determined. Vertex P is reached by starting at the origin and proceeding along half the length of the projection of vector $\boldsymbol{a}_2$ (see Table S10) in the *xy* plane. Thus it has Cartesian coordinates $\begin{pmatrix} X \\ Y \end{pmatrix} = \frac{1}{2}\begin{pmatrix} a_{2X} \\ a_{2Y} \end{pmatrix}$. Vertex Q is reached by starting at [0,1] and proceeding along half the length of the projection of vector $\boldsymbol{a}_1$. Thus it has Cartesian coordinates $\begin{pmatrix} X \\ Y \end{pmatrix} = \begin{pmatrix} 0 \\ a \end{pmatrix} + \frac{1}{2}\begin{pmatrix} a_{1X} \\ a_{1Y} \end{pmatrix}$. Similarly, vertex R has Cartesian coordinates $\begin{pmatrix} X \\ Y \end{pmatrix} = \begin{pmatrix} 0 \\ a \end{pmatrix} - \frac{1}{2}\begin{pmatrix} a_{2X} \\ a_{2Y} \end{pmatrix}$. Therefore parallelogram PQRS has area $A(PQRS) =$

$\frac{1}{4}\begin{vmatrix} -a_{2X} - a_{1X} & -a_{2Y} - a_{1Y} \\ a_{2X} - a_{1X} & -2a + a_{2Y} - a_{1Y} \end{vmatrix} = 2a^2x(\mathrm{O2})(1 - 2x(\mathrm{O2}))$ (S4.8). As the reference area for the degree of *z*-axis tilting = $a^2/4$, $\eta_A = 8x(\mathrm{O2})(1 - 2x(\mathrm{O2}))$ (S4.9).

N(tilt): 1. Parallelogram formation indicates $c^{\pm}$ tilting. Since there are no displacements ∥ z, the notation a^0a^0 applies. Assignation of these tilts does not require the octahedra to be regular.

$P4_2/nmc$ *z*=0

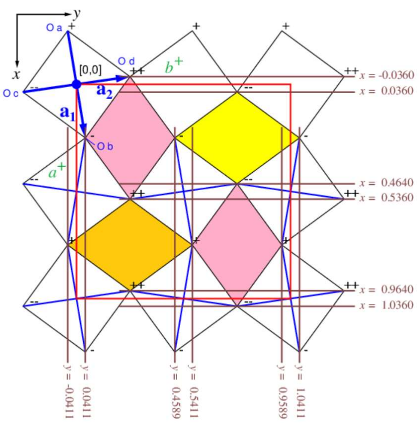

$P_{z=0.5}$

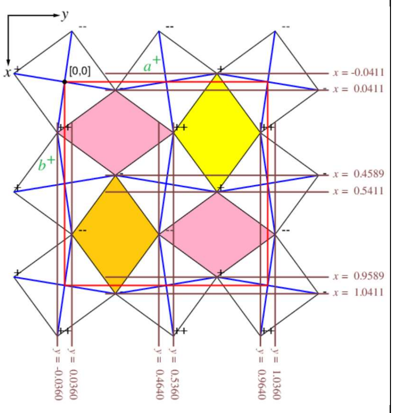

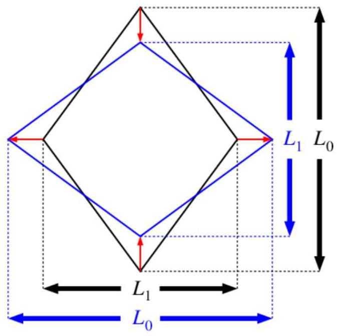

The AX_8 inner polyhedra are not right prisms in thus space group. Their form in *xy* projection is defined by two crossed rhombi, as may be inferred by superimposing the middle diagram above on the left-hand diagram. A plan-view is shown in the right-hand diagram, in which there are two defining parameters, L_0 and L_1, with $L_0 > L_1$. The area of each rhombus is equal to $\frac{1}{2}L_0L_1$. From the left-hand diagram it is inferred that $L_0 = 0.572a = \left(\frac{5}{2} - 2y(\mathrm{O3})\right)a$ and $L_1 = 0.4178a = \left(\frac{1}{2} - 2y(\mathrm{O2})\right)a$ for the pink-coloured rhombi. Similarly, $L_0 = 0.428a = \left(-\frac{3}{2} + 2y(\mathrm{O3})\right)a$ and $L_1 = 0.5822a = \left(\frac{1}{2} + 2y(\mathrm{O2})\right)a$ for the brown and the yellow-coloured rhombi. As the reference area for the degree of *z*-axis tilting = $a^2/8$, parameters φ_A reflecting degree of *z*-axis tilt are as follows.

Pink (4*d* cation): $\eta_{A,1} = 4\left(\frac{5}{2} - 2y(\mathrm{O3})\right)\left(\frac{1}{2} - 2y(\mathrm{O2})\right)$. (S4.10)

Brown (2*b* cation) and yellow (2*a* cation): $\eta_{A,2} = 4\left(-\frac{3}{2} + 2y(\mathrm{O3})\right)\left(\frac{1}{2} + 2y(\mathrm{O2})\right)$ (S4.11)

N(tilt): 3 or 2. Parallelogram formation and the two diagrams at *z*=0 and *z*=½ indicate c^- tilting. The two sets of equal and opposite displacements ∥ y, (+,-) and (++,--), lead to in-phase rotation aro& the *x* and *y* axes and the assignation a^+b^+. For regular octahedra the assignation a^+a^+ applies, signifying a reduction in N(tilt) from 3 to 2.

$R\overline{3}c$

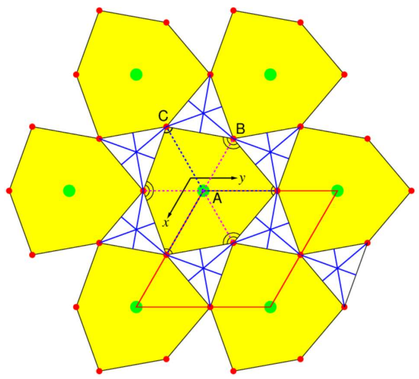

The diagram to the left shows a plane perpendicular to the *z*-axis in hexagonal axes at height *z*=¼. The A-ions (larger green circles) and X ions (smaller red circles) are coplanar. Six layers of this kind occur in the unit cell. Two further layers generated by rhomohedral translations [2/3, 1/3, 1/3] and [1/3, 2/3, 2/3]. Three further layers are interspersed by means of a *c* glide operation. AX_{12} polyhedra are formed from the irregular AX_6 yellow hexagons shown and layers above and below at *z*-heights of 1/12 and 5/12, these both contributing X3 triangles. Increasing irregularity of the AX_6 hexagons causes the volumes of the AX_{12} polyhedra to be reduced relative to the BX_6

octahedral volumes. $A(\text{hexagon}) = 6A(ABC)$. Further, without z-axis tilting, $A(ABC)$ would be one eighth of the *xy*-area of the unit cell $=a^2\sqrt{3}/16$. Inspection of the diagram reveals that lengths AB and AC are equal to $a(1 - x(\text{O}))$ and $ax(\text{O})$, respectively. Thus $A(ABC) = \frac{1}{2}a^2x(\text{O})(1 - x(\text{O}))\sin 60° = \frac{\sqrt{3}}{4}a^2x(\text{O})(1 - x(\text{O}))$ and $A(\text{hexagon}) = \frac{3\sqrt{3}}{2}a^2x(\text{O})(1 - x(\text{O}))$. (S4.12). The corresponding parameter for the degree of *z*-axis tilting is given by $\varphi_A = A(\text{hexagon})/\frac{3\sqrt{3}}{8}a^2 = 4x(\text{O})(1 - x(\text{O}))$. (S4.13). In the absence of tilting, *i.e.* when $x(\text{O}) = 1/2$, $\eta_A = 1$.

N(tilt): 1. The rotation of the octahedral triangles around the *z*-axis corresponds to a single tilt-system denoted $a^-a^-a^-$ with respect to pseudocubic axes.

References

Darlington, C. N. W. & Knight, K.S. (1999). *Acta Cryst.* B**55**, 24–30.

Fu, W.T., Visser, D. & Ijdo, D. J. W. (2005). *Solid State Commun.* **134**, 647–652.

Hahn, T. (1995). Editor. *International Tables for Crystallography*, Vol. A, *Space-Group Symmetry*. Dordrecht: Kluwer.

Mitchell, R.H., Burns, P.C., Knight, K.S., Howard, C.J. & Chakhmouradian, A.R. (2014). *Phys. Chem. Miner.* **41**, 393–401.

Williams, D.E. (1971). *Acta Cryst.* A**27**, 452–455.

Yang, J. (2008). *Acta Cryst.* B**64**, 281–286.

Yashima, M. & Ali, R. (2009). *Solid State Ion.* **180**, 120–126.

Zagorac, J., Zagorac, D., Zarubica, A., Schön, J.C., Djuris, K. & Matovic, B. (2014). *Acta Cryst.* B**70**, 809–819.
